# Adaptation and evaluation of a digital dialectical behaviour therapy for youth at clinical high risk for psychosis: A protocol for a feasibility randomized controlled trial

**DOI:** 10.1371/journal.pone.0339163

**Published:** 2025-12-23

**Authors:** Thea Lynne Hedemann, Yun Lu, Sofia Campitelli, Lisa D. Hawke, Nelson Shen, Sarah Saperia, Brett D. M. Jones, Gillian Strudwick, Chelsey R. Wilks, Wei Wang, Marco Solmi, Michael Grossman, Muhammad Ishrat Husain, Nicole Kozloff, George Foussias, Muhammad Omair Husain

**Affiliations:** 1 Campbell Family Mental Health Research Institute, Centre for Addiction and Mental Health, Toronto, Ontario, Canada; 2 Temerty Faculty of Medicine, Department of Psychiatry, University of Toronto, Toronto, Ontario, Canada; 3 Institute of Health Policy, Management and Evaluation, University of Toronto, Toronto, Ontario, Canada; 4 University of Washington, St. Louis, Missouri, United States of America; 5 University of Ottawa, Ottawa, Ontario, Canada; 6 University Health Network, Toronto, Ontario, Canada; PLOS: Public Library of Science, UNITED KINGDOM OF GREAT BRITAIN AND NORTHERN IRELAND

## Abstract

**Background:**

Youth at clinical high risk (CHR) for psychosis often experience emotional dysregulation, psychiatric symptoms, substance use, suicidality, and functional impairment. Dialectical behaviour therapy (DBT) is an evidence-based intervention that improves emotion regulation, clinical outcomes, and functioning across psychiatric populations. Digital adaptations (d-DBT) may enhance accessibility and engagement for CHR youth, but acceptability and potential benefits in this group are unknown.

**Objective:**

To adapt d-DBT for CHR youth and evaluate the acceptability of delivering it to this population, as well as the feasibility of a larger-scale clinical trial.

**Methods:**

This mixed-methods clinical trial has two phases. In Phase 1, d-DBT will be adapted for CHR youth in collaboration with a lived-experience youth advisory group. In Phase 2, an assessor-masked randomized controlled trial will compare d-DBT (n = 30) with treatment as usual (n = 30). The intervention consists of eight weekly modules, with primary outcomes assessing acceptability, usability, and trial feasibility. Secondary outcomes include changes in emotional dysregulation, psychiatric symptoms, substance use, suicidality, and functioning.

**Conclusions:**

We anticipate that d-DBT will be acceptable to CHR youth and that conducting a larger trial will be feasible. Preliminary findings may demonstrate improvements in emotion regulation, psychiatric symptoms, suicidality, and functioning. Results will guide further refinement of the intervention and inform the design of a confirmatory clinical trial.

**Trial registration:**

ClinicalTrials.gov #NCT06928935

## Introduction

Psychotic disorders are severe and enduring conditions associated with substantial distress, disability, and excessive mortality [1,2]. Longer-term functional outcomes in psychotic disorders are influenced by duration of untreated psychosis, and interventions delivered at the earliest stages of illness can significantly impact functional trajectories in affected individuals [3,4]. The Clinical High Risk (CHR) for psychosis concept was introduced to identify individuals at higher risk of developing psychosis, to provide indicated interventions, and avert progression to more severe outcomes [5]. The clinical state of CHR is characterized by three syndromes: (i) attenuated psychotic symptoms; (ii) brief intermittent psychotic symptoms; or (iii) genetic risk combined with a functional decline [6]. For young people meeting the CHR criteria, the cumulative risk of transitioning to a psychotic disorder is estimated to be approximately 19% at 2-year follow-up, and as high as 36.5% at 10-year follow-up [4]. There is strong evidence from a large, prospectively obtained, help-seeking cohort that almost three quarters of CHR individuals have another clinical diagnosis of mental illness that warrants treatment [7]. CHR individuals who do not transition to psychosis experience persistent attenuated psychosis symptoms, distress, psychiatric comorbidity, impaired functioning, reduced quality of life, and elevated risk of suicide, above and beyond the risk of developing psychosis [8–11]. These findings call for shifting the focus of CHR interventions from the dichotomy of transition to psychosis, to a wider concept of therapeutic treatment to prioritize addressing comorbidity, social recovery and functioning.

**Fig 1:**
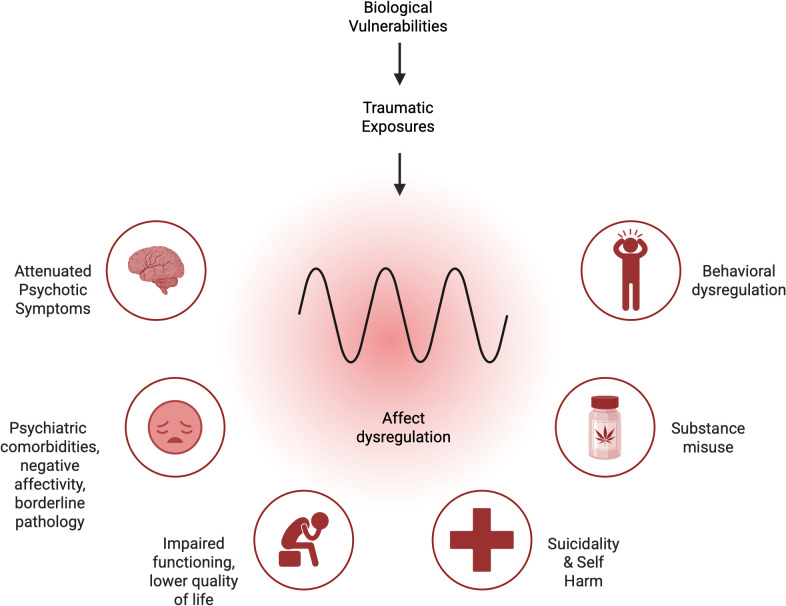
Illustration of the relationship between comorbidities in CHR population. *Created in BioRender. Hedemann,* ***T.*** *(2025)* *https://BioRender.com/0dexhpi.*

Young people with CHR experience high rates of psychiatric comorbidity, including anxiety, depression [8–10], personality disorder pathology [12], substance use [13], non-suicidal self-injury, suicidal ideation, and suicide attempts [11]. These comorbidities are associated with reduced quality of life, impaired functioning, and adverse outcomes [14]. One review found up to 80% of CHR patients had another mental illness, and nearly half had persistent psychosocial difficulties up to six years after first seeking help [9]. High rates of suicidality further underscore the need for timely assessment and intervention, with reported rates of 66% for recent suicidal ideation and 17.7% for lifetime suicide attempts [11]. Additionally, over 30% of CHR youth report cannabis use, which is associated with increased risk of psychosis and worsening of psychiatric comorbidities [15,16]. Borderline personality disorder (BPD) pathology is also common in CHR youth, with affective instability reported in 78% and self-harm in 74% [12,17]. Emotional dysregulation in this group is linked to poorer social functioning, more severe symptoms, and high rates of trauma exposure [18–21]. Given the strong overlap between CHR and BPD pathology—potentially driven by shared underlying mechanistic pathways including early life adversity [22–24]—targeting emotion dysregulation may be clinically impactful (Fig 1). However, standard CHR care rarely addresses BPD pathology and trauma, representing a critical treatment gap [25]. Interventions that promote emotion regulation and distress tolerance could improve functioning and reduce symptom severity in CHR youth [18].

Dialectical behavioural therapy (DBT) is a psychosocial intervention specifically designed for individuals at high risk for suicide who present with behavioural dysfunction related to emotion dysregulation [26]. Emotion regulation skills are central to DBT, an intervention based on a skills deficit model that views dysfunctional behaviour as either a consequence of dysregulated emotions or a maladaptive approach to emotion regulation. DBT skills training has been shown to be effective in reducing emotion dysregulation, suicidal behaviour, and substance use across psychiatric diagnoses [18,26]. To date, most clinical trials evaluating DBT have excluded individuals with psychotic disorders despite compelling evidence indicating that this population struggles with emotional dysregulation, impulsivity and is at an elevated risk of suicide. However, emerging lines of evidence support DBT as a feasible and acceptable intervention in individuals with psychosis along with encouraging clinical effects on emotion regulation and positive impacts on functioning [27]. To our knowledge, there are no clinical trials presently evaluating DBT interventions in the CHR population.

Access to mental health support and obtaining timely psychotherapy is challenging due to scarcity of resources [28]. Digital psychotherapy-based interventions present a discrete and socially acceptable solution for accessing care, particularly for youth who may be hesitant to seek mental health support [29]. Digital interventions can be used as stand-alone supports or to complement traditional care, including while individuals are on waitlists or through primary care. Evidence supports the feasibility and acceptability of delivering digital interventions to young people with CHR, alongside encouraging effects on clinical outcomes [30]. This study will adapt an existing, d-DBT intervention for use in young people at CHR for psychosis. d-DBT was initially developed by Wilks et al. (2018) for use in individuals with suicidality and severe alcohol use disorder [31] and later evaluated by our group as a transdiagnostic intervention for adult psychiatric inpatients with suicidality [32]. We hypothesize d-DBT can be clinically adapted and refined in a developmentally appropriate way for use in CHR youth. We also aim to evaluate the acceptability and usability of the d-DBT intervention and the feasibility of conducting a fully powered randomized controlled trial (RCT) of d-DBT in individuals at CHR for psychosis.

## Materials and methods

This is a multi-stage study employing qualitative and quantitative methods to adapt the d-DBT intervention and evaluate the acceptability of d-DBT to individuals with CHR, the feasibility of conducting a larger clinical trial and the preliminary efficacy in improving clinical and functional outcomes. The SPIRIT recommendations were followed in the reporting of this protocol (S1 File). The study has two distinct phases:

### Phase 1: Adaptation of d-DBT through engagement with youth

This phase will focus on the adaptation of d-DBT to be tailored to the needs and priorities of CHR youth through direct consultation with youth with lived-experience. Six youth with lived experience of CHR will participate in 16–24 structured sessions led by two Youth Engagement Specialists (YES). The YES will present the d-DBT model, walk through the content of each session and gain feedback from the advisory group on developmental- and diagnostic-relevant adaptations, explore attitudes and beliefs about the support needs of CHR youth, impact of illness and priorities/preferences for delivery to inform developmentally appropriate interventions. We will adhere to the Best Practice Guidelines for the Engagement of People with Lived Experience in Mental Health and Substance Use Research and recognized frameworks for engagement [33–35]. The content of the d-DBT intervention will be systematically presented to the youth through presentations and they will have hands-on experience of engaging with the prototype of the d-DBT application before its eventual build. Youth feedback will be solicited on many aspects of the content, including language, examples of skills, activities for skill development, app functionality, barriers and facilitators of use, and graphic design. Youth feedback on content of d-DBT intervention, usability, and engagement features will be shared with our multidisciplinary co-design team. The team will iteratively make changes to the intervention and bring them back to the youth for further feedback and confirmation. In making the intervention youth-friendly, there will be a balance between enhancing acceptability and maintaining fidelity to the core components of DBT. Phase 1 will complete following the adaptation of the intervention and the development of the digital platform. A service design approach will also be used to ensure platform alignment between the users, technologies, and clinical workflow [36].

### Phase 2: Pilot preliminary testing of d-DBT

d-DBT will be pilot tested for acceptability, feasibility of conducting a larger clinical trial and preliminary efficacy in an assessor masked randomized controlled clinical trial. Clinical and functional assessments will be performed at baseline once participants are enrolled in the study. Participants in the experimental arm will then receive 8 weeks of the d-DBT intervention and the control group will continue to receive standard care. Clinical and functional assessments will be repeated at the end of the 8-week intervention.

Feasibility and acceptability of d-DBT will also be assessed through semi-structured interviews with the participants after completion of trial. Semi-structured interviews will be conducted to obtain views of d-DBT, treatment target preferences, and experiences of assessment schedules. We will invite participants who completed the trial and participants who chose to withdraw from the study. Interview data will also be collected on experiences participating, technical experience, overall perception of the program, and barriers/facilitators to participation. The interview guide will be informed by established digital mental health intervention evaluation frameworks, along with input from the YAG [37]. Preliminary clinical efficacy of the d-DBT intervention will be informed by comparing changes in clinical and functional measures between the intervention and control arm at the end of the pilot study.

### Participants

Participants will be recruited from the Clinical High Risk (CHR) for Psychosis Clinical Service at the Centre for Addiction and Mental Health, which receives referrals from emergency departments, psychiatrists and primary care providers across Ontario, Canada. The service completes on average 100 new patient consultations annually.

#### Inclusion criteria.

1. Be 16–29 years old.
2. Be able to provide informed written consent to study participation.
3. Meets CHR criteria for a psychosis risk syndrome based on the Structured Interview for Psychosis Risk Syndromes (SIPS) [38] within the past 3 years.

#### Exclusion criteria.

1. Diagnostic and Statistical Manual of Mental Disorders (DSM-5) diagnosis of psychotic disorder (e.g., schizophrenia spectrum disorder, mood disorder with psychotic features)
2. Prior diagnosis of intellectual disability
3. Prior diagnosis of a severe developmental disorder
4. Acute suicidality requiring immediate life-saving intervention (i.e., inpatient psychiatric care).
5. Receiving any additional psychotherapy interventions or structured digital mental health support during the study period.

### Interventions

#### d-DBT intervention.

This d-DBT informed intervention was originally developed for individuals with suicidality, emotion dysregulation, and alcohol use disorder [31]. Our group has adapted and evaluated d-DBT for delivery as a transdiagnostic intervention for acute psychiatric inpatients with suicidality [32]. D-DBT will be tailored to the needs and priorities of CHR youth, for example, including specific modifications to address cannabis use—the most common substance in this population [13]. D-DBT includes eight modules covering mindfulness, emotion regulation, distress tolerance, and substance use. Each 30–50 minute module includes brief videos, key point summaries, guided skill practice, and homework assignments to practice skills introduced in each module. Each session includes 2–3 new DBT skills. Each new skill is first introduced via a 3–7-minute video segment. After the video, participants are directed to a “Key Point” page in which they are presented with the salient aspects from the video. Finally, participants engage in the skill through an interactive and guided practice. At the end of each session, participants select homework assignments. Homework assignments are intended to be completed prior to the next module commencing. At the end of each session, there will be a link to a brief survey where participants confirm completion of the module. A digital navigator will check-in with the participants via phone or videoconference (patient preference) to trouble-shoot any technical issues related to the intervention.

### Comparator

#### Treatment as usual.

All participants will continue with standard outpatient care, including routine healthcare provider appointments and medication management if indicated. There is limited access to case management and psychosocial supports within the Clinical High Risk Service at CAMH. Enrolled participants will be asked not to engage in concurrent psychosocial interventions for the duration of the trial. Any medication changes will be documented.

### Outcome measures

Demographic information, medical comorbidities, and current medication use will be collected at baseline. Psychiatric diagnoses will be confirmed using the Structured Clinical Interview for DSM-5 (SCID-5) and CHR characterization with the Structured Interview for Psychosis-risk Syndromes (SIPS) [38]. All clinical assessments will be conducted at baseline and post-intervention (8 weeks). Clinician-rated measures will be completed by trained research assistants who are blinded to participant treatmentallocation.

Primary outcomes:

1. Feasibility of conducting a confirmatory clinical trial will be informed by recruitment of proposed sample (n = 60), retention, and completeness of modules. Completeness of modules will be assessed using a brief survey after every module and participants will receive weekly virtual check-ins with a digital navigator to help with trouble shooting and enhance engagement. The platform will also track each participant’s progress. Participants’ ability to complete the clinical assessment schedule will also serve as a feasibility indicator.
2. Acceptability will be informed by the Client Satisfaction Questionnaire (CSQ-8) [39] and the System Usability Scale (SUS) [40].
3. Semi-structured interviews at the end of the intervention will also provide feasibility and acceptability data.

Secondary Outcomes (completed at baseline and 8 weeks):

1. Global Functioning: Social and Role Scales [41].
2. Psychosis spectrum symptoms: SIPS [38]; PRIME-Revised [42]
3. Mood and anxiety: The State-Trait Anxiety Inventory [43], Calgary Depression Scale for Schizophrenia [44].
4. Emotion Regulation: Brief Difficulties in Emotion Regulation Scale (DERS-16) [45].
5. Substance Use: Adolescent Alcohol and Drug Involvement Scale (AADIS); The Timeline Follow Back (TLFB) [46]; The Cannabis Use Disorders Identification Test-Revised (CUDIT-R) [47]; Daily Sessions, Frequency, Age of Onset and Quantity of Cannabis Use Inventory (DFAQ-CU) [48]
6. Borderline personality dimensions: BSL-23 [49].
7. Resiliency: Connor-Davidson Resilience Scale; CD-RISC 25 [50].
8. Suicidal ideation: Columbia-Suicide Severity Rating Scale [51].
9. MATRICS Consensus Cognitive Battery (MCCB) [52]
10. DBT ways of coping checklist [53].

#### Schedule of events.

A detailed schedule of enrolment, intervention delivery, and outcome assessments is shown in Table 1. Time points include enrolment, baseline, weekly intervention delivery, post-treatment assessment, and follow-up qualitative interviews.

**Table 1:** SPIRIT schedule of enrolment, interventions, and assessment.

	Screening	Baseline	d-DBT Intervention (8 weeks)	Follow up Visit
**TIMEPOINT**	***-t***_***1***_	**0**	**T1**	**T2**
**ENROLMENT:**				
Informed consent	X			
Inclusion/Exclusion Determination	X			
Randomization		X		
**INTERVENTION:**				
d-DBT program			X	
Treatment as usual (control)			X	
**ASSESSEMENTS:**				
SCID for the DSM-5	X			
SIPS	X			X
Prevention through Risk Identification, Management and Education Screen-Revised		X		X
Connor-Davidson Resilience Scale		X		X
Brief Difficulties in Emotion Regulation Scale (DERS-16)		X		X
Borderline personality dimensions: BSL-23		X		X
Calgary Depression Scale for Schizophrenia		X		X
The State-Trait Anxiety Inventory		X		X
Global Functioning: Social and Role Scales		X		X
Timeline Follow Back (TLFB)		X		X
Adolescent Alcohol and Drug Involvement Scale (AADIS)		X		X
The Cannabis Use Disorders Identification Test-Revised (CUDIT-R)		X		X
The Daily Sessions, Frequency, Age of Onset and Quantity of Cannabis Use Inventory (DFAQ-CU)		X		X
Columbia-Suicide Severity Rating Scale		X		X
MATRICS Consensus Cognitive Battery (MCCB)		X		X
DBT ways of coping checklist to measure DBT-skills use				X
Complete Case Report Forms (CRFs)	X	X	X	X
Client Satisfaction Questionnaire				X
System Usability Scale (SUS)				X
Adverse Event Log	X	X	X	X
Qualitative Interviews				X

#### Sample size justification and quantitative analysis plans.

We aim to recruit 60 CHR participants, with a focus on diversity across genders, ethnic origins, age, and other sociodemographic characteristics. The sample size was informed by published literature, which suggests 24–70 participants for pilot trials [54–56] and accounts for potential drop outs (~20%). The proposed sample size will provide reasonably reliable quantitative estimates for the targeted feasibility measures and preliminary data on clinical efficacy. The margins of error for 95% confidence intervals (CI) are ± 8.9% for recruitment rate, ± 10.1% for adherence/retention/attrition, and ±8.5% for completeness of outcomes. For secondary outcomes, the minimum detectable effect size is 0.72 (between-group Cohen’s *d*) and the margin of error for 95% CI is ± 0.57 SD.

Data will be analyzed principally using descriptive statistics to assess rates of recruitment, retention, intervention fidelity, engagement, and treatment response. Distributional characteristics of the outcome measures will be assessed for ceiling and floor effects and rates and patterns of missing values. Exploratory trial outcomes including impact of d-DBT on emotional regulation, psychiatric symptoms and functioning will be analyzed using (generalized) linear mixed-effects models with time (baseline, post-treatment), group (d-DBT vs control), time by treatment interaction as the primary predictor. The model will also include covariates to account for pre-treatment clinical characteristics and/or key demographics. The model form will be determined by the distribution features of the outcomes of interest (e.g., continuous, binary or count-type). The efficacy outcome will be impact of d-DBT on functioning, via the Global Functioning: Social and Role Scales. Our prior work with youth has informed us that functioning is more relevant to them than symptom severity [57]. Given the small sample size, appropriate to a feasibility trial, we will also calculate point and confidence estimate of effect sizes (Cohen’s *d*). Effect sizes will help with the estimation of the sample size needed for a larger scale randomized controlled trial in the future. Both ITT analysis and per-protocol/completers-only analysis will be conducted to gain insight of estimated efficacy of different subgroups of participants. Full information maximum likelihood approach will be adopted to deal with missing data under the missing-at-random assumption. Diagnostic analysis will be conducted to evaluate the soundness of the statistical assumptions carried by the methods. While the analytic approach may be complex for the small sample size, it will offer guidance for the analysis plan of the full-scaled trial.

#### Randomization.

Participants will be randomized in a 1:1 ratio to either the intervention or control group. The randomization schedule will be created by the trial statistician. Research assistants at the study site will obtain informed consent and enroll participants. Once enrolled, participants will be assigned to one of the two treatment conditions. Outcome assessors will be blinded to the treatment allocation.

#### Qualitative analysis plans.

The sample size for the qualitative component has been determined by the principle of saturation [58]; we anticipate that 15 post-intervention interviews will need to be conducted [59]. Participants will be recruited at completion of the clinical trial. Views of the d-DBT intervention, treatment target preferences, and experiences of assessment schedules will be obtained in individual, semi-structured interviews. Interview data will also be collected on usability of the virtual platform, experiences participating remotely/technical experience, overall perception of the program, and barriers/facilitators to participation. An interview guide will be developed from existing literature, together with feedback from the YAG, and will consist of key questions with prompts to enquire further. Interviews will be transcribed verbatim and analyzed using the five stages of framework analysis [60]. A pragmatic worldview will guide the analytic process, reflecting the study goal of informing real-world improvements to the intervention design and implementation [61]. To ensure rigor, an audit trail will be maintained to foster dependability of the process. A team-based approach will promote confirmability and credibility, including discussing emerging themes and involvement of the YAG in refining the codes and themes. Consensus of the final theoretical framework, reflexivity, and the impact of the researchers on recruitment, data collection and analysis will also be considered [62].

#### Fidelity.

d-DBT will be delivered virtually in a self-led program. This eliminates the concern of inter-practitioner variability in delivering psychotherapy. There will be weekly check-ins with our research staff who will act as ‘digital navigators’ and provide non-clinical support around application use which may include application troubleshooting, guidance through application exercises, reminders, and encouragement to support engagement [63]. They will have a semi-structured script which will explore if participants have been able to complete modules, explore barriers, and help answer basic questions about the content of the modules.

#### Anticipated timeline.

**Fig 2:**
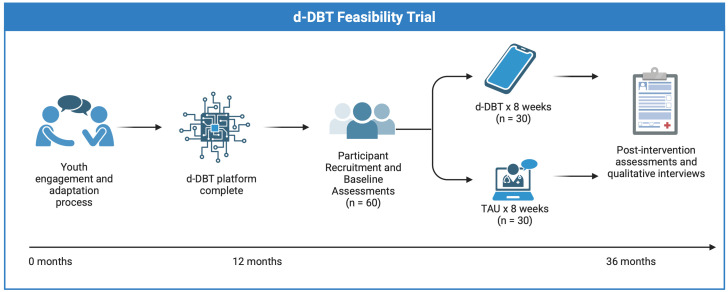
Study flow diagram. *Created in BioRender. Hedemann,* ***T.*** *(2025)* *https://BioRender.com/mrz1psp.*

This study was registered on ClinicalTrials.gov in March 2025 (#NCT06928935). As of May 2025, Phase 1 of the study which focuses on the adaptation of the digital DBT (d-DBT) platform is underway (Fig 2). Co-design sessions with the YAG are ongoing, with iterative feedback informing content and design modifications, as well as engagement strategies. The adaptation of the digital platform and the digital build is expected to be completed by December 2025. Participant recruitment will begin in January 2026. We aim to enroll 60 participants by December 2027, which is expected to be feasible based on our recruitment for other psychosocial intervention trials for CHR youth [64]. Clinical data collection and post-intervention assessments will be completed by early 2028. Qualitative interviews will be conducted after participants complete the clinical trial (Phase 2). Findings from the study will be reported following CONSORT guidelines for pilot and feasibility trials as well as the “Co-authoring and reporting on lived experience engagement in mental health and/or substance research” guidelines [65,66].

## Discussion

CHR youth experience high rates of psychiatric comorbidity, distress, impaired functioning, and increased risk of self-harm and suicide [8–12,14]. Early intervention during this high-risk stage is critical to improving long-term outcomes [3,4]. Yet access to appropriate mental health care remains a widespread challenge, with long wait times, limited availability of specialized services, and stigma acting as significant barriers [28]. Moreover, there are few evidence-based psychosocial treatments specifically designed for CHR youth that are developmentally appropriate, trauma-informed and focus on addressing psychiatric comorbidity and poor functioning [25].

This study responds to these gaps by adapting and evaluating a d-DBT intervention tailored for CHR youth. Co-developed with lived experience youth, the intervention is grounded in emotion regulation theory and addresses common comorbidities such as borderline pathology and substance use. By centering youth voices in the design process, the intervention aims to enhance engagement, relevance, and acceptability [33–35].

Digital platforms offer a discreet, scalable, and cost-effective solution to delivering evidence-based care to youth who might otherwise remain untreated. Remote delivery enables access across a range of contexts, including rural or underserved regions and settings with limited specialized mental health services. The self-guided nature of the program may further reduce barriers to participation, offering flexibility and privacy that align with youth preferences. We anticipate that the intervention will be acceptable for CHR youth, that a larger confirmatory clinical trial will be feasible and that preliminary findings may suggest clinical benefits in improving emotion regulation, reducing psychiatric symptoms, and enhancing functioning. Results from this pilot study will inform further refinement of the intervention and guide the design of a fully powered randomized controlled trial aimed at improving early intervention strategies for youth at risk of psychosis.

### Ethical considerations and safety

This study was approved by the Centre for Addiction and Mental Health (CAMH) Research Ethics Board (REB #2025/007, Version 2.0, S2 File). All study procedures will adhere to institutional guidelines, the Tri-Council Policy Statement (TCPS-2), and the ethical principles of the Declaration of Helsinki (1975, revised 2008). All research staff will complete institutional ethics training and certification in Good Clinical Practice (GCP) prior to any participant facing activity. Standard operating procedures for confidentiality, data security, and consent will be followed. Identifiable data will be securely stored, and access restricted to authorized personnel.

Participation is voluntary and participants may withdraw at any time without impact on their standard care. Participants in the control arm will be offered the d-DBT intervention after study completion. There are no anticipated risks to the participants as the intervention is a ‘light-touch’ psychosocial intervention. A risk management protocol will be used to safely manage acute suicidality or psychosis, and the digital navigator will conduct weekly check-ins to monitor for concerns. Although unblinding is not anticipated, the trial manager may unblind in cases where safety or protocol integrity requires it. Identifiable data will be securely stored and accessed only by authorized personnel.

## Supporting information

S1 File SPIRIT Checklist for clinical trial protocol. (PDF)

S2 File REB approved protocol. (DOCX)

## Abbreviations

DBT: Dialectical behaviour therapy
d-DBT: digital dialectical behaviour therapy
CHR: clinical high risk, RCT, randomized controlled trial, YES, youth engagement specialist.
