# Supplementary material for: Adaptation and evaluation of a digital dialectical behaviour therapy for youth at clinical high risk for psychosis: A protocol for a feasibility randomized controlled trial
Source: PLoS One. 2025 Dec 23;20(12):e0339163. doi: 10.1371/journal.pone.0339163 (PMC12725739; doi:10.1371/journal.pone.0339163)
Supplement: S2 File — (DOCX) [file pone.0339163.s002.docx]

**Digital dialectical behavioural therapy (d-DBT) for youth at Clinical High Risk (CHR) for psychosis: an exploratory multi-methods study**

**Protocol Identifying Number (if applicable): *2025/007 (modified version of 2023/010)***

**Principal Investigator:** **Dr. Omair Husain**

**Version Number: 1.3**

**Version Date: 13-Mar-2025**

**Table of Contents**

STATEMENT OF COMPLIANCE 4

1.0 INTRODUCTION 5

1.1 Background 5

1.2 Study Intervention 9

1.3 Clinical Data to Date 10

1.4 Risks/Benefits 10

2.0 CLINICAL TRIAL OBJECTIVES 11

2.1 Specific Aim 1 11

2.2 Specific Aim 2 11

2.3 Specific Aim 3 11

3.0 CLINICAL TRIAL DESIGN 11

3.1 Overall Design 11

3.2 Patient Engagement 12

3.3 Quantitative methods 12

3.4 Qualitative methods 12

3.5 Outcome Assessments 13

4.0 PARTICIPANT SELECTION AND WITHDRAWAL 14

4.1 Target Population 14

4.2 Participant Recruitment and Screening 14

4.3 Eligibility Criteria 16

4.3.1 Inclusion Criteria 16

4.3.2 Exclusion Criteria 16

4.4 Participant Withdrawal Criteria 16

5.0 STUDY INTERVENTION 17

5.1 Description 17

5.2 Treatment Regimen 17

5.3 Method for Assigning Participants to Treatment Groups 19

5.4 Administration of Study Intervention 19

5.5 Participant Compliance Monitoring 19

5.6 Concomitant Therapy 19

6.0 RESEARCH PROCEDURES 19

6.1 Research Visits 19

6.2 Schedule of Events 23

7.0 STATISTICAL PLAN 24

7.1 Sample Size and Power for quantitative methods 24

7.2 Sample size for qualitative methods: 24

7.3 Statistical Methods 24

8.0 SAFETY AND ADVERSE EVENTS 25

9.0 DATA MANAGEMENT, RETENTION AND INTEGRITY 25

10.0 ETHICAL CONSIDERATIONS 27

10.1 Research Ethics Board (REB) Approval 27

10.2 Informed Consent Process & Documentation 27

11.0 PRIVACY AND CONFIDENTIALITY 28

12.0 REFERENCES 29

# STATEMENT OF COMPLIANCE

This clinical trial will be carried out in accordance with the following:

- International Conference on Harmonisation Good Clinical Practice (ICH GCP)
- Tri-Council Policy Statement 2018 (TCPS 2)
- Personal Health Information Protection Act (PHIPA), 2004; Chapter 3 Schedule A (PHIPA) and applicable regulations
- Institutional and REB policies and procedures


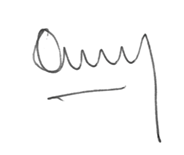
 13-Mar-2025

_______________________________ _____________________________

Signature of PI Date

# INTRODUCTION

## Background

**Clinical high risk (CHR) for psychosis**

Psychotic disorders are severe and enduring conditions associated with substantial distress, disability, and excessive mortality (1, 2). Longer-term functional outcomes in psychotic disorders are influenced by duration of untreated psychosis, and interventions delivered at the earliest stages of illness can significantly impact functional trajectories in affected individuals (3, 4). The Clinical High Risk (CHR) for psychosis concept was introduced to identify individuals at higher risk of developing psychosis, to provide indicated interventions, and avert progression to more severe outcomes (5). The clinical state of CHR is characterized by three syndromes: (i) attenuated psychotic symptoms; (ii) brief intermittent psychotic symptoms; or (iii) genetic risk combined with a functional decline (6). The CHR construct is increasingly being recognized as a distinct disorder, and a new diagnostic category of attenuated psychosis syndrome was considered in DSM-5 as a discrete clinical condition. For young people meeting the CHR criteria, the cumulative risk of transitioning to a psychotic disorder is estimated to be approximately 19% at 2-year follow-up, and as high as 36.5% at 10-year follow-up (4). In large cohorts of people with CHR, approximately a third will remit spontaneously and require no further treatment (7) (8) but more commonly people will develop mood disorders, anxiety and substance use disorders (7, 9). There is strong evidence from a large, prospectively obtained, help-seeking cohort that almost three quarters of CHR have another clinical diagnosis of mental illness that warrants treatment (10). Longitudinal research indicates that CHR individuals who do not transition to psychosis experience persistent attenuated psychosis symptoms, distress, psychiatric comorbidity, impaired functioning, reduced quality of life, and elevated risk of suicide, above and beyond the risk of developing psychosis (11-13) (14). These findings call for the re-conceptualization of CHR as a distinct syndrome, the importance of which extends beyond being a predictor for psychotic disorder. Shifting the focus of CHR interventions from the dichotomy of transition to psychosis, to a wider concept of therapeutic treatment to prioritize social recovery and functioning is a logical step towards prioritizing patient-centered goals.

**Comorbidities in CHR**

Young people with CHR are highly susceptible to psychiatric comorbidities, with high prevalence of anxiety and depression (11-13), personality disorder pathology (15), substance use (16), suicidal ideation and attempted suicide (14). Psychiatric comorbidities in CHR are associated with reduced quality of life, impaired functioning, and negative clinical outcomes (17). This is consistent with a recent systematic review that found **up to 80% of CHR patients had another diagnosable mental illness, and almost half experienced poor psychosocial functioning up to six years after first seeking help** (12). For these reasons, appropriate strategies addressing the unique mental health needs of young people with CHR are warranted.

*Personality disorder pathology and trauma*

Personality disorder pathology is prevalent in CHR individuals with **an estimated 45% of affected individuals having co-morbid personality disorder pathology** (18). The most prevalent personality disorder pathology identified in CHR youth is borderline personality disorder (BPD) pathology (25%) (15, 19). In one study, almost all CHR youth endorsed at least one feature of BPD, and the most reported was affective instability (77.9 %) (19). Compared with healthy controls, individuals with CHR experience elevated emotional reactivity and emotional dysregulation (20). Greater emotional reactivity and emotion dysregulation in CHR individuals is associated with lower social functioning and greater negative symptom severity (20). Emotional dysregulation in CHR is also associated with high prevalence of self-harm (73.8%) (19). BPD pathology in CHR individuals is associated with more severe attenuated psychotic symptoms (21), anxiety and depressive symptoms, and a history of trauma (22). There may be a shared etiological pathway that underlies CHR and BPD given that childhood trauma and adversity are empirically associated with both conditions (23). It is suggested that the relationship between adverse childhood experiences and attenuated psychotic symptoms may be mediated by BPD symptomatology (24). The association between childhood trauma and psychosis is well established (25) and a large proportion of CHR individuals (87%) report trauma exposure (26), highlighting the need for trauma-focused and trauma-informed interventions in these at-risk populations (27). However, standard CHR care does not address BPD pathology, leaving a critical gap in the treatment of these individuals. Trauma-informed therapeutic interventions designed to reduce emotion reactivity and enhance emotional regulation may be effective in reducing clinical symptomatology and improving global functioning in CHR (20).

*Mood and anxiety disorders*

Evidence from a large cohort of CHR youth indicated that approximately **70% had one or more mood or anxiety disorder diagnosis** (18). Meta-analysis demonstrates that the comorbid diagnosis of depressive and anxiety disorders is 41% and 15% respectively (10). Depression and anxiety co-morbidity in CHR is linked to increased suicidality, self-harm and poorer functioning, necessitating prompt assessment and treatment (7). In young people (aged 15-29 years) suicide is the second leading cause of death worldwide (28) and psychosis-like symptoms are associated not only with comorbid mental health problems and poor functioning but also with suicide (29). Fewer depressive symptoms are associated with higher rates of remission in CHR suggesting that the absence of depression or treatment of depression may be a good prognostic indicator in this population (30). Focusing attention on treatment of co-morbid mood and anxiety disorders in CHR may represent a strategy to improve clinical and functional outcomes in affected individuals and has the potential to achieve a profound public health impact (31).

*Self-harm and suicide*

Self-harm and suicidality are commonly seen in youth with CHR. A meta-analysis of suicidal behaviour in **CHR individuals reported a high prevalence rate of 66% for recent suicidal ideation, 49% for lifetime self-harm and 17.7% for lifetime suicide attempts** (14). Suicidality was found to be associated with depressive symptoms, childhood adversities and trauma (14). This link has been demonstrated in individuals with psychosis as well as individuals with CHR (10). Suicide is a major public health concern across the world and individuals with CHR are particularly vulnerable to suicide, above and beyond the risk of psychosis. The high prevalence of suicidality in this population highlights the need for monitoring suicide risk and providing targeted suicide prevention interventions in CHR.

*Substance use*

CHR youth are vulnerable to high levels of substance use, in particular cannabis, which not only elevates risk of transition to psychosis, but also has the potential to exacerbate existing psychiatric comorbidity (32). Meta-analysis has demonstrated that current cannabis abuse or dependence is strongly associated with elevated risk of transition to psychosis (33). The North American Prodrome Longitudinal Study 2 (NAPLS-2) reported that the prevalence of current cannabis use in a large sample of CHR youth was over 30% (34). Substance use disorders are often considered maladaptive coping strategies to alleviate depression, anxiety, and other comorbidities in youth (35). It is possible that a shared underlying psychopathology predisposes individuals with CHR to developing psychosis and cannabis abuse or dependence disorder (33). Traumatic life experiences may be the underlying predisposing factor underlying vulnerability to substance use and psychotic disorders further supporting the indications for trauma-informed psychosocial interventions to address these comorbidities in individuals with CHR (36, 37).

**Gaps in interventions for CHR**

Conventional CHR intervention trials have focused on transition to psychosis, psychotic symptoms, and associated distress with little emphasis on functional outcomes across diagnoses (38, 39). Nonetheless, meta-analyses support interventions delivered during CHR in effectively delaying transition to a full-blown psychotic illness, reducing psychotic-like symptoms at 12-months and improving functional outcomes (40) (38). While these findings are important, studies of discrete interventions do not reflect real-world complexity where multifaceted treatments that are adapted to individual needs are indicated (41). We would argue that broader multicomponent interventions are required for individuals with CHR, encompassing biological and psychosocial aspects as well as personalizing them to the needs of the individual in their sociocultural and developmental context. In contrast to later-stage illness, during CHR there is uncertainty about illness trajectory that favors a ‘light touch’ approach and transitioning to individualized, multicomponent treatments is more consistent with heterogeneity seen in this population. The evidence described above supports that most individuals with CHR have another clinical diagnosis of mental illness that warrants treatment and several experience poor psychosocial functioning. For these reasons, developing and testing multi-component interventions that address complex intersecting needs in developmentally informed ways, including youth and family engaged intervention development and evaluation projects, reflecting the dynamic nature of youth development are needed for young people with CHR.

Although the relationship between comorbidities in CHR remains complex, **emotional dysregulation may represent a therapeutic target and common link among the multiple comorbidities in CHR**. Emotional dysregulation encompasses a set of maladaptive responses including high emotional reactivity, threat sensitivity, difficulties processing and expressing emotions (42). Emotional dysregulation is a component of several mental disorders including anxiety, depression, personality disorders, psychosis, substance use disorders and is associated with elevated risk of self-harm and suicidality (42-44). Emotional dysregulation manifests across the psychosis spectrum (43) and is associated with severity and frequency of psychotic symptoms in individuals with established psychotic disorders (45). Emotional dysregulation also affects individuals with CHR (20, 43, 46, 47) and is associated with lower social functioning and greater negative symptom severity (46). Individuals with emotion dysregulation often have patterns of behaviour dysregulation, including substance misuse, self-harm, and suicidal behaviours. These behaviours are often the result of poor emotional distress tolerance, maladaptive emotional regulation strategies (48) and problem-solving functions employed to cope with negative emotions (44). Emotional dysregulation represents a potential treatment target for negative affect, impaired functioning, suicidality, self-harm, and substance misuse in individuals with CHR. Current CHR treatment strategies include psychosocial interventions like cognitive behaviour therapy, which are indicated for treating comorbid depression and anxiety (49). **To our knowledge there are no published clinical trials of interventions that target the additional comorbidities,** which may contribute to impaired functioning and disability in **CHR youth.** Interventions that enhance adaptive emotional regulation and distress tolerance skills could prove beneficial to CHR youth in reducing clinical symptomatology and improving global functioning (20).

**Dialectical behavioural therapy (DBT)**

Dialectical behavioural therapy (DBT) is a trauma-informed psychosocial intervention specifically designed for individuals at high risk for suicide who present with behavioural dysfunction related to emotion dysregulation (50). Emotion regulation skills are central to DBT, an intervention based on a skills deficit model that views dysfunctional behaviour as either a consequence of dysregulated emotions or a maladaptive approach to emotion regulation. DBT skills training has been shown to be effective in reducing emotion dysregulation, suicidal behaviour, and substance use across psychiatric diagnoses (20, 50). As a transdiagnostic intervention in non-BPD populations, DBT has been found to be acceptable and shown efficacy in improving symptoms of depression (51), reducing anxiety and depression in people with eating disorders (52), reducing depressive symptoms and suicidal ideation in youth with bipolar disorder (53), as well as improving emotional dysregulation and decreasing anxiety in a transdiagnostic sample (54). Evidence also supports DBT as a feasible and acceptable intervention in individuals with psychosis along with encouraging clinical effects on emotion regulation and positive impacts on functioning (55). Based on the well-established theory and substantial existing evidence, DBT may offer promise as an intervention that can potentially address multiple psychiatric comorbidities, reduce distress, increase self-efficacy, and improve functioning in the CHR population. **However, to our knowledge there are no clinical trials presently of DBT interventions in CHR population**.

**Digital DBT in CHR**

Access to mental health support and obtaining timely psychotherapy is challenging in Ontario due to scarcity of resources. In Ontario, publicly funded psychotherapy is primarily provided by primary care physicians (PCPs) with psychotherapy-focused practices and psychiatrists, who are unable to meet the psychotherapy demands of Ontarians (56). Higher-income individuals are more likely to access private psychotherapy services, resulting in income-based inequalities in access to psychotherapy (57). Additional barriers include long wait times, geographic constraints, cultural and language barriers, as well as stigma (58). These challenges are particularly concerning for the CHR population, who need timely preventative care. Digital psychotherapy-based interventions present a discrete and socially acceptable solution for accessing care, particularly for youth who may be hesitant to seek mental health support. It is often low cost, does not require intensive training of psychotherapists, provides patient-centered flexibility, and is accessible regardless of geographic location (59). Evidence supports the feasibility and acceptability of delivering digital interventions to young people with CHR, alongside encouraging effects on clinical outcomes (60). Digital DBT (d-DBT) is a web-based platform developed by our group at the Centre for Addiction and Mental Health (CAMH), Canada’s largest academic mental health center. In a PSI-funded feasibility trial, d-DBT was feasible to administer and acceptable to patients admitted to CAMH with suicidal ideation (manuscript in preparation). d-DBT a psychosocial intervention delivered virtually through a web-based portal, which can be adapted to provide a framework to address the psychosocial and mental health needs of individuals with CHR, adopting a patient-centered and trauma-informed approach. Data from pilot trials support the acceptability and feasibility of this treatment model (61).

This current grant proposal details the design and delivery of a comprehensive, evidence-based, ‘ready-to-go’ psychosocial and mental health intervention, to effectively enhance mental health and support functional recovery for individuals with CHR. Failure to provide adequate and timely mental health support to CHR individuals is a missed opportunity to avert progression to more severe outcomes, at which stage affected individuals are more likely to engage with mental health services. Treatment strategies catered to the evolving needs of the CHR population that aim to improve clinical outcomes have the potential to significantly reduce the personal and societal burden attributed to mental illness in Ontario and across Canada. Given the high prevalence of personality pathology, substance use, suicidality, and poor functioning within the CHR population and the pivotal developmental stages CHR presents, effective treatment is likely to deliver substantial economic and social gains, reducing disability and enhancing productivity. Digital DBT is a psychosocial intervention delivered virtually through a web-based portal, which provides a framework to address the psychosocial and mental health needs of individuals, adopting a patient-centred and trauma-informed approach. This study has the potential to inform the implementation of evidence-based interventions in Ontario and elsewhere. Leveraging technology to deliver digital DBT (d-DBT) offers a cost-effective and scalable platform to deliver evidence-based interventions. Virtual delivery of care offers a discrete and socially acceptable way of accessing care, especially for youth who may be hesitant to seek mental health support.

## Study Intervention

We plan to adapt and use a d-DBT skills intervention developed by Linehan et al. in the USA for individuals with suicidality and severe alcohol use (61). d-DBT was acceptable, safe, and effective in reducing suicidal ideation, substance use, and emotion dysregulation (61). Our group have recently adapted d-DBT and delivered this as a trans-diagnostic intervention to individuals hospitalized to psychiatric inpatient departments. The d-DBT intervention consists of eight modules delivered over 8-weeks. Adaptation in the present study will focus substance use to include cannabis, as this is the most used recreational substance in the CHR population (16). Skills like “dialectical drinking” will be adapted to focus on cannabis use and substance use more broadly. A youth engagement specialist will lead a youth advisory council who will co-design the d-DBT intervention.

## Clinical Data to Date

DBT has been shown to be effective in reducing emotion dysregulation, suicidal behaviour, and substance use (20, 50). As a transdiagnostic intervention in non-BPD populations, DBT has been found to be acceptable and shown preliminary efficacy in decreasing emotion dysregulation and decreasing anxiety (54). The pilot study on d-DBT in individuals with alcohol use disorders have demonstrated feasibility in terms of recruitment, acceptability, and implementation (61). However, d-DBT has never been adapted and applied to the CHR population.

## Risks/Benefits

There are no anticipated risks to the participants as the intervention is a ‘light-touch’ psychosocial intervention. It is incumbent upon the research team to identify subjects who require treatment and actively assist them in getting it. Anyone identified with acute psychosis or at acute risk of suicide will be assisted to immediately contact local mental health professionals. If required, the research team will accompany the participant to the local mental health service provider. We have developed a standard operating procedure for risk management that will be adhered to throughout the study. All assessments will be completed in accessible, private, and appropriate venues that suit the needs and preferences of participants. The appointments will be scheduled at times convenient to participants, considering education, household commitments and employment commitments. Participants will not be exposed to a risk of physical and mental harm that is greater than that typically encountered in normal life and the recruitment materials will direct participants to relevant supports if participation raises any concerns. The Digital Navigator will connect with participants weekly, if there are any concerns about risk they will follow our risk management protocol.

# CLINICAL TRIAL OBJECTIVES

## Specific Aim 1

To adapt an existing, d-DBT intervention for use in young people at CHR for psychosis. **H1:** d-DBT can be clinically adapted and refined in a developmentally appropriate way for virtual use among CHR individuals.

## Specific Aim 2

To evaluate the acceptability of the d-DBT invention and the feasibility of conducting a fully powered randomized controlled trial (RCT) of d-DBT in individuals at CHR for psychosis. **H2:** d-DBT will be acceptable to CHR individuals as demonstrated by qualitative feedback, attendance rates of d-DBT sessions, the Client Satisfaction Questionnaire (CSQ) and the System Usability Scale (SUS). d-DBT for CHR individuals will be feasible as demonstrated by our ability to recruit our desired sample of 60 participants and retain 80% of those recruited by the end of the 8-week study period. Participants’ ability to complete the clinical assessment schedule will also serve as a feasibility indicator.

## Specific Aim 3

To assess the preliminary efficacy of d-DBT in improving functioning and reducing psychiatric comorbidities in individuals at CHR for psychosis in an assessor-masked preliminary RCT clinical trial. **H3**: Individuals with CHR receiving d-DBT will demonstrate enhanced resiliency, reduced depression and anxiety, personality disorder pathology, improved emotional regulation scores, reduced substance use, reduced suicidal ideation, improved functioning and cognitive performance as a result of treatment compared to CHR individuals receiving treatment as usual.

# CLINICAL TRIAL DESIGN

## Overall Design

This is a multi-methods study employing qualitative and quantitative methods to adapt the d-DBT intervention and test the feasibility, and acceptability of d-DBT in individuals with CHR. d-DBT will first be adapted to CHR individuals in collaboration with a youth advisory group. Then an assessor-masked pilot RCT, where d-DBT will be delivered over 8-weeks, will be conducted. Sixty participants are planned to be enrolled in the RCT, 30 in the d-DBT group and 30 in the treatment as usual group. Participants in the d-DBT group will continue with their standard outpatient care. Outcome measures will be completed at study entry and post-treatment. For the qualitative component of the study, 15 post-intervention semi-structured interviews will be conducted.

## Patient Engagement

We will recruit four-six youth with lived-experience to form a youth advisory group to advise on the study design, help build interview guides and advise on iterative adaptations of d-DBT. We will present the d-DBT model, explore attitudes and beliefs about the support needs of CHR youth, experiences of accessing and engaging with treatments, perceptions of psychosocial interventions, impact of illness and priorities/preferences for delivery to inform developmentally appropriate interventions. The group will also advise on the development/adaptations of the digital platform that the intervention will be delivered on. The youth advisory group will also help develop consent and demographic forms to ensure a youth friendly approach to the project. We will discuss study findings with the youth advisory group and collaborate with them in knowledge translation through co-authorship of study results.

## Quantitative methods

Assessor-masked pilot RCT comparing the adapted d-DBT intervention to standard outpatient care. Participants in the d-DBT arm will receive the intervention over 8-weeks. Measures will be completed at study entry and repeated immediately post-treatment at 8-weeks. Clinician rated assessments will be completed with a trained research assistant who will be unaware of treatment allocation.

## Qualitative methods

The sample size for the qualitative component has been determined by the principle of saturation (62); we anticipate that 15 post-intervention interviews will need to be conducted (63). Participants will have the opportunity to consent to the qualitative interview during the initial informed consent discussion at the time of recruitment. The interview would be conducted virtually or in person. We will invite participants who completed the trial and participants who chose to withdraw from the study. Views of the d-DBT intervention, treatment target preferences, and experiences of assessment schedules will be obtained in individual, semi-structured interviews at intervention completion. Interview data will also be collected on usability of the virtual platform, experiences participating remotely/technical experience, overall perception of the program, and barriers/facilitators to participation. An interview guide will be developed from existing literature, together with feedback from the youth advisory group, and will consist of key questions with prompts to enquire further. All interview data will be transcribed verbatim. Data will be analyzed using the five stages of framework analysis (64). Rigor will be ensured by maintaining an audit trail to foster dependability of the process. Confirmability and credibility will be assured by a team approach to data analysis and discussion of the emerging themes. Consensus of the final theoretical framework, reflexivity, and the impact of the researchers on recruitment, data collection and analysis will also be considered (65).

## Outcome Assessments

**Measures/Assessment Tools:** Demographic and medical comorbidity assessments, and current medication use will be gathered at baseline. Measures will be completed at study entry and repeated immediately post-intervention at 8-weeks. Clinician rated assessments will be completed with a trained research assistant. Any changes to medication will be recorded at intervention completion. Psychiatric diagnoses will be confirmed using the Structured Clinical Interview for DSM-5 (SCID-5) and Structured Interview for Psychosis-risk Syndromes (SIPS) at baseline. Clinical assessments will be completed at baseline and 8-weeks.

Primary outcomes:

1. Feasibility outcomes will include recruitment (60 participants), retention, and completeness of modules. Completeness of modules will be assessed using a brief survey after every module and participants will receive weekly virtual check ins to help with trouble shooting and enhance engagement. Participants’ ability to complete the clinical assessment schedule will also serve as a feasibility indicator.
2. Acceptability will be informed by the Client Satisfaction Questionnaire (CSQ-8) (66). Semi-structured interviews at the end of the intervention will also provide feasibility and acceptability data.
3. System Usability Scale (SUS) (67) - assesses whether the intervention is serviceable for its intended purpose. The SUS is a reliable tool for measuring the usability. It consists of a 10-item questionnaire with five response options for respondents; from Strongly agree to Strongly disagree.

Secondary Outcomes:

1. Global Functioning: Social and Role Scales (68).
2. Psychosis spectrum symptoms: SIPS (69); PRIME-Revised (70)
3. Mood and anxiety: The State-Trait Anxiety Inventory (71), Calgary Depression Scale for Schizophrenia (72).
4. Emotion Regulation: Brief Difficulties in Emotion Regulation Scale (DERS-16) (73).
5. Substance Use: Adolescent Alcohol and Drug Involvement Scale The Timeline Follow Back (TLFB) method for measuring quantity of cannabis, alcohol, and tobacco use in the past 7 days (74); The Cannabis Use Disorders Identification Test-Revised (CUDIT-R) (75);Daily Sessions, Frequency, Age of Onset and Quantity of Cannabis Use Inventory (DFAQ-CU) (76)
6. Borderline personality dimensions: BSL-23 (77).
7. Resiliency: Connor-Davidson Resilience Scale; CD-RISC (78).
8. Suicidal ideation: Columbia-Suicide Severity Rating Scale (79).
9. Cognition: The MATRICS Consensus Cognitive Battery (MCCB) (80)

**Fidelity**

d-DBT will be delivered virtually in a self-led program. This eliminates the concern of inter-practitioner variability in delivering psychotherapy. There will be weekly check-ins with our research staff who will act as ‘digital navigators’ and provide non-clinical support around application use which may include application troubleshooting, guidance through application exercises, reminders, and encouragement to support engagement (81). They will have a semi-structured script which will explore if participants have been able to complete modules, explore barriers, and help answer basic questions about the content of the modules. We will also use the DBT ways of coping checklist to measure DBT-skills use (82).

# PARTICIPANT SELECTION AND WITHDRAWAL

## Target Population

We will recruit 60 CHR participants, with a focus on diversity across genders, ethnic origins, age, and other sociodemographic characteristics. All participants will be assessed at baseline and 8-weeks. We will recruit youth (age 16 – 29 years). The sample size was informed by published literature, which suggests 24-70 participants for pilot trials (83-85).

## Participant Recruitment and Screening

Study participants will be recruited from the Slaight Family Centre for Youth in Transition (SFCYT) via two mechanisms: 1) the Slaight centralized clinical research recruitment strategy in SFCYT where a core team of clinical research staff identify and engage all patients receiving early psychosis services in clinical research, providing streamlined triaging of patients to appropriate studies; 2) the CAMH-wide Clinical Engagement and Research Recruitment (CLEARR) infrastructure that enables early identification and engagement in research for all new patients presenting for care at CAMH.

As part of the CLEARR process, a delegated, CLEARR approved Research Coordinator/Physician will identify potential participants and notify the research team and the participant’s clinician about the participant’s eligibility to participate in the study. The clinician will then ask the participant if they would be willing to meet with a study team member about participating. Only with participants’ agreement will they be approached.

The CLEARR coordinator/physician will access personal health information (PHI) in participants’ health records to determine potentially eligible participants for the study.

In case of referrals from clinics, recruitment for the study will be initiated by the clinical team who is treating the potential study participant. The treating physician/clinical care team will not obtain consent. They may identify potential research participants and obtain verbal permission from these potential participants for a member of the research team to approach them. Potential participants who indicate an interest in hearing more about the study and provide verbal consent to be contacted will be contacted by a member of the research team who will engage them in an informed consent process.

We will also contact former research participants who participated in previous studies or are currently participating in parallel studies and agreed to hear about future research opportunities and consented to be re-contacted. To avoid cold calling, the participants will be contacted by someone from the research team that the former participant was involved with.

Research personnel will contact participants according to their preferred mode of communication (email, telephone, SMS text), which will be recorded in the pre-screening/subject log. In the case of text messaging, personnel will only contact participants to schedule or remind participants of upcoming appointments.

All patients will be given the option to participate. Participation in the study is voluntary. The decision to participate will not affect patients’ receipt of treatment or clinical services. Participants will be informed that they have the option of terminating their participation at any time, without consequence and that no new data will be collected on them.

Participants will be recruited through the Slaight Centre for Youth in Transition’s centralized recruitment process. All subjects will be pre-screened with the Slaight REB approved “pre-screen form” that is implemented across all SFCYT studies to allow for alignment and data sharing and recruitment; this data will be entered into a password protected and restricted pre-screen tracking database. We conduct case review meetings twice a week to review recruitment and to identify new patients who may be eligible for research studies. Our centralized recruitment strategy allows us to ‘triage’ and ‘group’ participants into non-competing research studies to encourage referrals among PIs.

Recruitment will also be enhanced by the Toronto Adolescent and Youth (TAY) Cohort study (PI; Dr A Voineskos) that is recruiting 3000 youth, and emerging adults, aged 11-24 years over 5-years. The TAY study offers rich phenotypic data and aims to investigate mechanisms of disease in young people presenting to mental health services. We expect to identify many individuals with CHR from the TAY Cohort study, where to date almost 50% of participants demonstrate clinically significant levels of psychosis spectrum symptoms.

Following provision of written informed consent, participants will be assessed for suitability for inclusion in the study based on the inclusion and exclusion criteria. If it is deemed necessary, research staff may review participants’ CAMH medical charts for scheduling purposes and/or to obtain additional information for ongoing eligibility determination and/or obtain other clinically relevant information for research purposes. We will also record information about the participant’s mental health from their charts that we are unable to obtain during their interviews. The investigator or research staff may also request information from the participant’s treating psychiatrist (CAMH) after obtaining consent for release of personal health information to determine eligibility and obtain clinically relevant information for research purposes.

Study participants will receive a small honorarium to reimburse them for the time and inconvenience incurred during participation in this study. For initial assessments, post-intervention assessments and post-intervention qualitative interviews, participants will receive up to $60 for these encounters.

## Eligibility Criteria

### Inclusion Criteria

The participant must meet all the inclusion criteria to eligible for this study:

1. Be 16-29 years old.
2. Being competent and willing to consent to study participation.
3. Meets CHR criteria for a psychosis risk syndrome based on the Structured Interview for Psychosis Risk Syndromes (SIPS) within the past 3 years.

### Exclusion Criteria

An individual who meets any of the following criteria will be excluded from participation in this study:

1. Diagnostic and Statistical Manual of Mental Disorders (DSM-5) diagnosis of psychotic disorder (e.g., schizophrenia spectrum disorder, mood disorder with psychotic features)
2. Diagnosis of intellectual disability
3. Severe developmental disorder
4. Acute suicidality requiring immediate life-saving intervention (i.e., inpatient psychiatric care).
5. Receiving any additional psychotherapy interventions or structured digital mental health support during the study period.

## Participant Withdrawal Criteria

Participants are advised in the consent form that they are free to withdraw from the study at any time without prejudice and may be withdrawn at the Investigators discretion.

The study participation will also be terminated should participants develop or are found to have any condition that might compromise safety (e.g., unstable vitals). Other reasons for withdrawing individual participants from the study may include one of the following:

- Major protocol violation
- Participant lost to follow-up.
- Withdrawal of consent
- Any participant may be discontinued from the study at the discretion of the investigators if it is deemed to be in the best interest of the participant.

The reason for participant discontinuation or withdrawal from the study will be recorded within the participant’s research record, and/or legal health record.

# STUDY INTERVENTION

## Description

We plan to adapt and evaluate a d-DBT skills intervention developed by Linehan et al. in the USA for individuals with suicidality and severe alcohol use (61). d-DBT was acceptable, safe, and effective in reducing suicidal ideation, substance use, and emotion dysregulation (61). Our group have recently adapted d-DBT and delivered this as a trans-diagnostic intervention to individuals hospitalized to psychiatric inpatient departments. The d-DBT intervention consists of eight modules delivered over 8-weeks. Adaptation in the present study will focus substance use to include cannabis, as this is the most used recreational substance in the CHR population (16). Skills like “dialectical drinking” will be adapted to focus on cannabis use and substance use more broadly (Table 1). Our youth advisory council will be consulted on the youth friendly language and provide input on adaptation of the intervention.

## Treatment Regimen

The first two modules will focus on mindfulness skills (“What” and “How” skills), the following two modules are focused on reducing problematic substance use (“Dialectical Drinking,” “Clear Mind,” “Community Reinforcement,” and “Burning Bridges”), the subsequent three modules are focused *
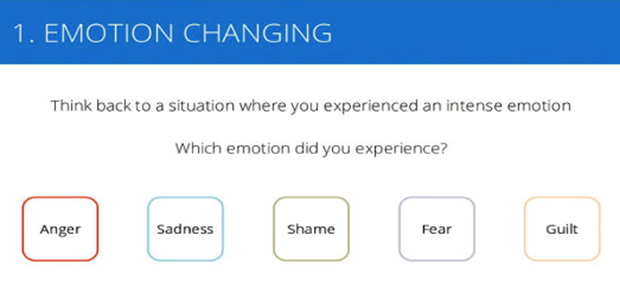
*on increasing emotion regulation strategies (“Model of Emotions,” “Check the Facts,” “Opposite Action,” “Problem Solving,” “Building Mastery,” and “Cope Ahead”), and finally, the last module includes skills on tolerating distress (“TIP” & “Distract”) (Table 1).


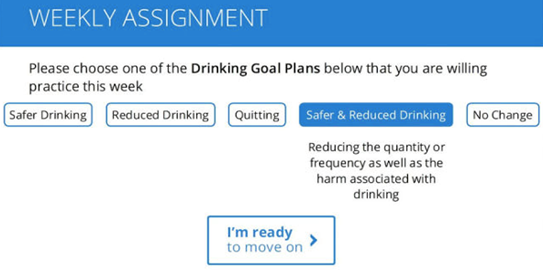
Each session includes 2–3 new DBT skills. Each new skill will first be introduced via a 3 to7-minute video segment delivered by Marsha Linehan. After the video, participants will be directed to a “Key Point” page in which they will be presented with the salient aspects from the video. Finally, participants will engage in the skill through an interactive and guided practice. At the end of each session, participants will select a homework practice. These homework assignments will be embedded in the newly adapted d-DBT platform and are intended to be completed prior to the next module. Each session lasts approximately 30–50 minutes. At the end of each session, there will be a link to a brief survey where participants will confirm their completion of the module. Every week, a member of the research staff will check-in with the participants via phone or video platform (patient preference) to enhance adherence and trouble-shoot any issues related to the intervention.

Table 1. Modules and the main skills included in d-DBT.

| Module 1-2 Mindfulness skills: | “What” skills: these are ‘what’ you are doing while practicing mindfulness. They are ‘observe’, ‘describe’ and ‘participate.  “How” skills: these are ‘how’ you practice mindfulness. They are non-judgmentally, one-mindfully, and effectively. |
| --- | --- |
| Module 3-4 Substance use skills: | Clear Mind: Clear mind represents the synthesis between clean mind and addict mind. In clear mind, you are clean, while at the same time you stay aware of the dangers of relapse and actively engage in behaviors to prevent a lapse or relapse  Burning Bridges: Burning bridges involves reducing your opportunities to engage in the addictive behavior. Basically, you're burning the bridge between you and people, places, or events in your life that encourage or facilitate addiction or maladaptive behaviors  Dialectical Drinking. The skill of dialectical drinking describes the abstinence violation effect, goal setting, pros and cons of maintaining or changing their drinking behavior, as well as an assessment of motivation and confidence to follow-through on their drinking goal. We plan to adapt this module to focus on cannabis use and substance use more broadly. |
| Module 5-7 Emotion regulation skills: | Model of Emotions: breaks down your emotional experience into 6 parts. Changing any one part can change the entire response.  Check the Facts: helps you change your emotional response and make healthier decisions as a result. Using check the facts, you can modify your response to a level that is appropriate for the situation, or respond with a more fitting emotion  Opposite Action: is a deliberate attempt to act opposite of your emotion urge. If your emotions are doing more harm than good, try acting opposite  Problem Solving: is a skill to do a behavioral analysis, assess what can be changed, brainstorm alternatives, and practice putting into action.  Building Mastery: A skill that helps improve self-esteem and confidence.  Cope Ahead: is skill about making a plan for ourselves for dealing with a stressful situation we will encounter in the future |
| Module 8 Distress Tolerance Skills | TIP: A distress tolerance skill that is an acronym that stands for Temperature, Intense Exercise, Paced Breathing, and Progressive Relaxation. TIP skills ask that we change our body chemistry to regain control of our emotions and behavioral responses.  Distract: a group of distraction techniques for distress tolerance |

Duration of treatment and follow up: The d-DBT intervention will be delivered over 8 weeks and participants in the experimental arm will be asked to complete 1 module per week. Measures will be completed at study entry and repeated immediately post-treatment at 8 weeks.

**Control Group Procedures**

Participants in the control group will continue to receive treatment as usual, which may consist of routine appointments with healthcare providers, medication management, and other supportive services as deemed necessary by their care team. Outcome measures will be collected at study entry and at 8-weeks for participants in the treatment as usual group.

## Method for Assigning Participants to Treatment Groups

Participants will be randomized by a randomization schedule designed by the trial statistician to one of the two groups, d-DBT versus treatment as usual.

## Administration of Study Intervention

The d-DBT will be delivered virtually with a self-led program. Please see section 3.5 for further details.

## Participant Compliance Monitoring

Poor engagement and attrition from digital interventions is an increasingly recognized challenge for interpreting and translating the findings from digital health research. Meta-analysis has reported that 26% of individuals dropout of digital interventions for depressive symptoms (39), whereas premature termination of in person psychotherapy is estimated at 20% (40). The addition of a human component reduces attrition by more than 50% (39), therefore this study intends to use digital navigators to troubleshoot technical issues and enhance therapeutic alliance (34). Digital navigators will complete brief virtual check-ins with participants every week during the intervention. This will help enhance retention and engagement and identify any potential barriers participants are having to complete their module.

## Concomitant Therapy

Patients participating in this study will continue to be eligible for and continue their standard care, which comprises an outpatient multidisciplinary team approach including, psychiatric follow up and potential medication management. Concomitant medication/therapies will be verified with research participants at each visit. Concomitant medication analysis will be conducted using multivariate analysis to control possible effects. Receiving any additional psychotherapy interventions or structured digital mental health support during the study period is an exclusion criterion for the study.

# RESEARCH PROCEDURES

## Research Visits

The d-DBT intervention will be delivered over 8-weeks. Measures will be completed at study entry and repeated immediately post-treatment at 8-weeks. Assessments will be completed with a trained research analyst.

When possible, assessments will be done virtually. Please see schedule of events below. After informed consent has been obtained; participants will complete screening assessments to determine eligibility; followed by baseline clinical, functional, and neuropsychological assessments. The clinical, functional, and psychological assessments will be repeated approximately 8-weeks after baseline. These assessments can be scheduled on the same day or separate days depending on participant preference. If participants completed overlapping clinical, cognitive, or functional assessments as part of their participation of any CAMH study they may be shared, with participant consent. Additionally, many clinics at CAMH use certain rating scales and collect demographic, educational, and cognitive information as part of routine clinical care. For study participants where this information has already been collected as part of routine clinical care, we will endeavor to utilize this already collected information rather than repeating these assessments with participants, if the participant agrees (based on participant consent). Sharing information and assessments will reduce participant burden both by shortening research assessment duration and reducing the stress that can occur with repeated questions on the same topics. Consent for this will be obtained as part of the informed consent procedures for this study.

**Clinical Assessments**

To reduce participant burden, where possible clinical assessments will not be repeated if administered in another study (as outlined above).

**Categorical Assessments**

The Structured Clinical Interview for DSM-5 (SCID-5) (86) will be used for all participants. The SCID is a semi-structured diagnostic interview designed to assist clinicians, researchers, and trainees in making reliable DSM-5 psychiatric diagnoses. Can be administered virtually. [Approximately 60-120 minutes to complete]

**Dimensional Assessments**

*Attenuated Psychosis Symptoms*

All participants will be administered the PRIME-Revised (70). This assessment is harmonized with existing measures already utilized in several studies and clinical services at CAMH to minimize participant/patient burden [5 minutes to complete].

CHR participants will be administered the Structured Interview for Prodromal Symptoms (SIPS) (69): The SIPS is a widely used structured interview for diagnosing a CHR syndrome for psychosis and cases of first episode psychosis. It contains a severity rating scale (the Scale Of Psychosis-risk Symptoms, or SOPS), a well-anchored Global Assessment of Functioning (GAF), the DSM-IV schizotypal personality disorder checklist, a brief assessment of the family history of psychosis, and the Criteria Of Psychosis-risk Syndromes (COPS) and Presence Of Psychosis Scale (POPS) and DSM-5 Attenuated Psychosis Syndrome criterion sets. The SIPS rules in/out the current and lifetime presence of psychosis and when psychosis has never been present diagnoses three CHR syndromes. Can be administered virtually. [120 minutes to complete]

*Mood Symptoms*

Calgary Depression Scale for Schizophrenia (CDSS)(72) is a 4-point Likert type scale (0, absent; 1, mild; 2, moderate; 3, severe) with nine items. It is a reliable scale, with high internal consistency (Cronbach’s alpha = 0.855), and the CDSS is suitable for evaluating symptoms of depression in people at clinical high risk for psychosis(87). Survey administration and can be done virtually. [10 minutes to complete]

*Anxiety Symptoms*

The State-Trait Anxiety Inventory (STAI)(71) is a psychological inventory consisting of 40 self-report items on a 4-point Likert scale. The STAI measures two types of anxiety – state anxiety and trait anxiety. Higher scores are positively correlated with higher levels of anxiety. Survey administration and can be done virtually. [5 minutes to complete]

*Borderline personality dimensions*

The Borderline Symptom List (BSL-23) (77) is a well-established self-rating instrument to assess the severity of borderline typical psychopathology. The BSL-23 assesses 23 feelings and experiences typically reported by BPD patients, refers to the last week and has a range from 0 = ‘not at all’ to 4 = ‘very strong. Survey administration and can be done virtually. [10 minutes to complete]

*Emotion Regulation*

The Difficulties in emotion regulation scale (DERS)(73) is a 36-item self-report measure of six facets of emotion regulation. Items are rated on a scale of 1 (“almost never [0–10%]”) to 5 (“almost always [91–100%]”). Higher scores indicate more difficulty in emotion regulation. [5 minutes to complete]

*Functioning*

Global Functioning: Social and Role Scales - brief clinician-administered measures of social and role functioning, respectively, administered as a semi-structured interview with detailed anchors for ratings that address functional difficulties typically experienced by youth at risk for psychosis (88). Can be administered virtually. [10 minutes to complete]

*Resiliency*

The Connor-Davidson Resilience Scale (CD-RISC)(78) is a 25-item self-report rating scale designed to assess resilience, with higher scores being an indicator of high resilience. Each item is rated on a 5-point scale ranging from not true at all or zero to true nearly all the time or four. The total possible scores range from 0–100. [5 minutes to complete]

*Substance use*

Adolescent Alcohol and Drug Involvement Scale (AADIS) is a clinician rated instrument which evaluates an adolescent’s use of alcohol and other drugs (89). [5-10 minutes to complete]

The Timeline Follow Back (TLFB) ((74)) –is a self-report method for substance use, and will be used to quantify the amount/frequency of alcohol, cannabis, and tobacco in the past 7 days. [5 minutes to complete]

The Cannabis Use Disorders Identification Test-Revised (CUDIT-R) is a self-report tool developed that aim to distinguish between different levels of cannabis use, cannabis use disorders and stage of change (75). It contains 8 items, two each from the domains of consumption, cannabis problems (abuse), dependence, and psychological features. [5 minutes to complete]

The Daily Sessions, Frequency, Age of Onset and Quantity of Cannabis Use Inventory (DFAQ-CU) is a self-report tool designed to assess key dimensions of cannabis use, including the number of daily use sessions, frequency of use over time, age of first use, and quantity consumed (76). Survey administration and can be done virtually [5 minutes to complete]

*Suicide Risk*

Columbia-Suicide Severity Rating Scale (C-SSRS)(79) is a semi-structured clinical.

interview that assesses suicidal ideation severity, suicidal ideation intensity, and suicidal

behavior. It is suited to use in youth at risk for psychosis. [10 minutes to complete]

*Neurocognitive Domains*

The MATRICS Consensus Cognitive Battery (MCCB) (80) is intended to provide a relatively brief evaluation of key cognitive domains relevant to schizophrenia and related disorders. The MCCB includes ten tests that assess seven cognitive domains: (1) speed of processing; (2) attention/vigilance; (3) working memory; (4) verbal learning; (5) visual learning; (6) reasoning and problem solving; and (7) social cognition. Requires in person administration for standardization. HVLT, Maze, and BVMT forms will be counterbalanced at baseline and follow-up. [60 minutes to complete]

**Acceptability**

1. Client Satisfaction Questionnaire (CSQ-8), a self-reported scale with 8 items that describes satisfaction with a health service (66);

2. System Usability Scale (SUS) (67): This is a self-report survey that determines whether the intervention is serviceable for its intended purpose. The SUS is a reliable tool for measuring the usability. It consists of a 10 item questionnaire with five response options for respondents; from Strongly agree to Strongly disagree.

## Schedule of Events

| **Procedures** | Screening | Baseline | d-DBT Intervention (8 weeks) | Follow up Visit |
| --- | --- | --- | --- | --- |
| Informed consent | X |  |  |  |
| Inclusion/Exclusion Determination | X |  |  |  |
| SCID for the DSM-5 | X |  |  |  |
| SIPS | X |  |  | X |
| Prevention through Risk Identification, Management and Education Screen-Revised |  | X |  | X |
| Connor-Davidson Resilience Scale |  | X |  | X |
| Brief Difficulties in Emotion Regulation Scale (DERS-16) |  | X |  | X |
| Borderline personality dimensions: BSL-23 |  | X |  | X |
| Calgary Depression Scale for Schizophrenia |  | X |  | X |
| The State-Trait Anxiety Inventory |  | X |  | X |
| Global Functioning: Social and Role Scales |  | X |  | X |
| Timeline Follow Back (TLFB) |  | X |  | X |
| Adolescent Alcohol and Drug Involvement Scale (AADIS) |  | X |  | X |
| The Cannabis Use Disorders Identification Test-Revised (CUDIT-R) |  | X |  | X |
| The Daily Sessions, Frequency, Age of Onset and Quantity of Cannabis Use Inventory (DFAQ-CU) |  | X |  | X |
| Columbia-Suicide Severity Rating Scale |  | X |  | X |
| MATRICS Consensus Cognitive Battery (MCCB) |  | X |  | X |
| d-DBT program |  |  | X |  |
| DBT ways of coping checklist to measure DBT-skills use |  |  |  | X |
| Complete Case Report Forms (CRFs) | X | X | X | X |
| Client Satisfaction Questionnaire |  |  |  | X |
| System Usability Scale (SUS) |  |  |  | X |
| Adverse Event Log | X | X | X | X |
| Qualitative Interview |  |  |  | X |

# STATISTICAL PLAN

## Sample Size and Power for quantitative methods

Prior digital intervention feasibility trials in CHR have found that a minimum of 14 participants is sufficient to determine feasibility(90). The sample size was informed by published literature, which suggests 24-70 participants for pilot trials (83-85). We aim to recruit 60 participants in this 8-week pilot RCT to account for potential dropouts (~20%). The proposed sample size will provide reasonably reliable quantitative estimates for the targeted feasibility measures and preliminary data on clinical efficacy. The margins of error for 95% confidence intervals (CI) are ±8.9% for recruitment rate, ±10.1% for adherence/retention/attrition, and ±8.5% for completeness of outcomes. For Aim 3, the minimum detectable effect size is 0.72 (between-group Cohen’s d) and the margin of error for 95% CI is ±0.57 SD.

## Sample size for qualitative methods:

The sample size for the qualitative component has been determined by the principle of saturation (62); we anticipate that 15 post-intervention interviews will need to be conducted (63). Views of the d-DBT intervention, treatment target preferences, and experiences of assessment schedules will be obtained in individual, semi-structured interviews at intervention completion. Interview data will also be collected on usability of the virtual platform, experiences participating remotely/technical experience, overall perception of the program, and barriers/facilitators to participation. An interview guide will be developed from existing literature, together with feedback from the youth advisory group, and will consist of key questions with prompts to enquire further. All interview data will be transcribed verbatim. Data will be analyzed using the five stages of framework analysis (64). Rigor will be ensured by maintaining an audit trail to foster dependability of the process. Confirmability and credibility will be assured by a team approach to data analysis and discussion of the emerging themes. Consensus of the final theoretical framework, reflexivity, and the impact of the researchers on recruitment, data collection and analysis will also be considered (65).

## Statistical Methods

Data will be analyzed principally using descriptive statistics to assess rates of recruitment, retention, intervention fidelity, engagement, and treatment response. Distributional characteristics of the outcome measures will be assessed for ceiling and floor effects and rates and patterns of missing values. Exploratory trial outcomes including impact of d-DBT on emotional regulation, psychiatric symptoms and functioning will be analyzed using (generalized) linear mixed-effects models with time (baseline, post-treatment), group (d-DBT vs control), time by treatment interaction as the primary predictor. The model will also include covariates to account for pre-treatment clinical characteristics and/or key demographics. The model form will be determined by the distribution features of the outcomes of interest (e.g, continuous, binary or count-type). The efficacy outcome will be impact of d-DBT on functioning, via the Global Functioning: Social and Role Scales. Our prior work with youth has informed us that functioning is more relevant to them than symptom severity (91). Given the small sample size, appropriate to a feasibility trial, we will also calculate point and confidence estimate of effect sizes (Cohen’s *d*) Effect sizes will help with the estimation of the sample size needed for a larger scale confirmatory randomized control trial in the future. Both ITT analysis and per-protocol/completers-only analysis will be conducted to gain insight of estimated efficacy of different subgroups of participants. Full information maximum likelihood approach will be adopted to deal with missing data under the missing-at-random assumption. Diagnostic analysis will be conducted to evaluate the soundness of the statistical assumptions carried by the methods. While the analytic approach can be seen as too complex for the small sample size, it will offer guidance for the analysis plan of the full-scaled trial.

# SAFETY AND ADVERSE EVENTS

Adverse Events (AEs) and Serious Adverse Events (SAEs) will be documented and reported as mandated by current regulations. AEs will be assessed at each study visit. Any AEs will be followed up to resolution or appropriate resolution will be taken as per the direction of the QI. All AEs, whether reported by the participant or observed by study staff/investigators, will be recorded on the AE log along with a brief description, start date/resolution date and any action taken. The AE log will be initialed by the QI, who will make the determination on the seriousness, relationship, and expectedness of the AE to the investigational drug/study procedures. Where the event is deemed to meet CAMH REB reporting criteria, the QI will notify REB in accordance with CAMH REB’s reporting requirements and timelines.

# DATA MANAGEMENT, RETENTION AND INTEGRITY

The basic protection against risk in this study will be provided by Dr. Husain (study PI and QI). He will have primary responsibility for monitoring of participants during the entire time they participate in the study. The PI will meet weekly with study personnel to review accrued data, data confidentiality, and adherence to protocol design, recruitment, and participant complaints. During meetings, the study PI will also review the enrollment data, the accrual and integrity of clinical, and neurocognitive data, and any adverse event associated with the various components of the study. Based on these reports, we will determine if there has been any change in the benefit-to-risk ratio of the clinical and cognitive assessment components of the study.

Participant Study File (PSF) will be completed for each participant enrolled in the study. All information recorded on the PSFs for this study will be considered the participant’s source documentation. Source data from the PSF will be collected and documented on paper and/or using the “Electronic Data Capture” solution (REDCap). A participant screening and enrollment log, noting reasons for screen failure, where applicable, will be maintained for all participants.

Study data will be entered in a secure database (REDCap). At point-of-entry, data values will undergo consistency edits (e.g., ID validation, range verification, duplicate detection) and personnel will be required to correct errors. Data management staff will run logic error programs to check for accuracy and irregularities within and across data structures. Quality assurance checks will be conducted daily by the personnel, as well as biweekly by data management staff. To reduce the incidence of missing data during data acquisition, we will use several strategies available in REDCap, including marking data fields as required and use of the Data Quality module to run regular queries for ALL missing data. This will alert study personnel/PI of missing values and regular data audits using REDCap’s Data Resolution Workflow, allowing a data auditor to open queries (based on the Data Quality Module) for data entry personnel to respond to (and leaving an audit trail that can be tracked). For the relevant assessments, the study REDCap database will also retain form statuses to reflect whether an assessment was collected in-person or virtually.

The hard copies of data will be stored in a locked filing cabinet in a locked office to further protect participant anonymity. Data auditing, entry, and quality control will be carried out routinely. Regularly scheduled communication between the study team and the PI will clarify any inconsistencies and ambiguities in the data. Additional communication will be conducted as needed.

Interview audio recordings will be securely stored on CAMH secure servers, which are protected from unauthorized access by passwords. During audio recording interviews, use of direct identifiers will be avoided. Should any personal information appear in the transcripts, that information will be censored. Quotes from interviews may be used in dissemination of the research findings, including conference presentations and peer reviewed journal articles, but no information will be included that might identify participants. Recordings, coded interviews will be securely stored on CAMH servers for 10 years, as per CAMH Data Retention guidelines. Steps will be taken to ensure the confidentiality of participants’ information.

All data pertaining to a participant’s involvement in this study will be coded and stored securely (in locked offices or secure database/server). This information will only be accessible to the research team. In unusual cases, a participant’s research records may be released in response to a court order. If the research team learns that a participant or someone with whom the participant is involved with is in serious danger or harm, an investigator will inform the appropriate agencies as per legal or regulatory requirements.

During the duration of the study, data and all appropriate documentation will be maintained according to current regulations and stored for a minimum of 10 years after the completion of the study.

# ETHICAL CONSIDERATIONS

## Research Ethics Board (REB) Approval

Research Ethics Board (REB) approval will be obtained prior to beginning any research-specific procedures. Following initial ethics approval, ongoing ethical approval will be maintained, and the clinical trial will undergo REB review at least annually, in accordance with regulatory and REB requirements. The clinical trial will be conducted in accordance with the REB-approved study documents and the determinations (including any limitations) of the REB, and in compliance with REB requirements.

Whenever new information becomes available that may be relevant to participant consent, a consent form and/or consent for addendum will be presented to the REB for review and approval prior to its use. Any revised written information will receive REB approval prior to use.

## Informed Consent Process & Documentation

Potential participants who are capable to consent will be approached and provided with information and pertinent details of the study following the ‘Telephone/Email script to schedule study visits’ document.

The decision to participate will in no way influence or restrict treatment services, and the participant is free to withdraw at any time with no negative consequences. The individual obtaining the informed consent will review any points about which the potential participant is unclear, and the participant will be invited to ask questions. Our research staff are carefully trained in strategies for interacting with people with severe mental illness, including speaking slowly and clearly, stopping to summarize frequently, and providing time for questions. They are all supervised by senior staff members.

Participant will be assessed for competency to provide informed consent and will review the consent document with the study staff. Informed consent will be obtained in-person.

Participant will be assessed for competency to provide informed consent and will review the consent document with the study staff. The research staff will go through the informed consent form (ICF) and will explain the study in detail and answer any questions the participant has. After reading the consent, but before obtaining a signature, all participants who express willingness to provide consent will be queried about the consent form to verify that the participant has demonstrated comprehension of the study and consent document and is competent to provide informed consent. Following the participant signature, the person conducting the consent discussion will also sign the consent attestation page, alongside the participant. Physical copies of the signed ICFs and Attestation pages will be provided to the participants.

# PRIVACY AND CONFIDENTIALITY

The Investigator will preserve the confidentiality of participants taking part in the study as mandated by current regulation. All personal study participant data collected and processed for the purposes of this study will be managed by the investigators and their staff. Adequate precautions to ensure the confidentiality of those data, and in accordance with applicable national and local laws and regulations on personal data protection will be used (in accordance with CAMH policies, PHIPA, Tri-Council Policy Statement (TCPS2) and the International Conference on Harmonization Guideline for Good Clinical Practice (ICH GCP) requirements).

There is a potential risk of breach of confidentiality that is inherent in all research protocols. Breach of confidentiality will be minimized by the staff who will maintain research data (identified only by participant code number not related to name or date of birth) in separate charts and a dedicated password protected electronic database. A list of participant names, their ID numbers, and information about how they can be reached will be kept in a separate locked cabinet with access only to study personnel authorized by the PI. Procedures have been established, and will be followed, to minimize the risk of breach of confidentiality. Procedures to maintain confidentially include: (1) formal training sessions for all research personnel emphasizing the importance of confidentiality; (2) specific procedures developed to protect participants’ confidentiality, and (3) formal mechanisms limiting access to information that can link data to individual participants. All information obtained from participants will be kept as confidential as possible. Computer based files/data will be entered into password-secured databases and paper-based files will be stored in a secure location. These data will only be accessible to personnel involved in the study and they will abide by confidentiality regulations of the REB. The ethics committee granting approval to this study will be granted direct access to the study participants’ original medical records for verification of study procedures and/or data, without violating the confidentiality of the participants, to the extent permitted by the law and regulations.

Research data gathered as part of this study may be shared and provided to other investigators working with the study team for the purpose of data sharing. If participants are enrolled in multiple studies, their research data will be shared across studies to reduce participant burden and avoid duplication of procedures. Only investigators/research team affiliated with the study team will have access to secured files and/or research data and will be well-informed regarding the protection of participants’ rights to confidentiality.

Furthermore, investigators collaborating with the study team will have access to the research data collected during the study for the purposes conducting secondary analyses about mental illnesses, such as ASD, depressive disorders, psychotic disorders, bipolar disorders, anxiety disorders, sleep disorders, etc. These data will be de-identified and not contain any PHI.

Participants will not be identified by name in any publication or presentations at meetings of research results. Results will be published as group data without the use of characteristics that would identify individual participants.

All study-related records will be kept for at least 10 years according to current regulations.

# REFERENCES

1. Vos T, Barber RM, Bell B, Bertozzi-Villa A, Biryukov S, Bolliger I, et al. Global, regional, and national incidence, prevalence, and years lived with disability for 301 acute and chronic diseases and injuries in 188 countries, 1990–2013: a systematic analysis for the Global Burden of Disease Study 2013. The Lancet. 2015;386(9995):743-800.

2. Simon GE, Stewart C, Yarborough BJ, Lynch F, Coleman KJ, Beck A, et al. Mortality Rates After the First Diagnosis of Psychotic Disorder in Adolescents and Young Adults. JAMA Psychiatry. 2018;75(3):254-60.

3. Marshall M, Lewis S, Lockwood A, Drake R, Jones P, Croudace T. Association between duration of untreated psychosis and outcome in cohorts of first-episode patients: a systematic review. Archives of general psychiatry. 2005;62(9):975-83.

4. Fusar-Poli P, De Micheli A, Signorini L, Baldwin H, Salazar de Pablo G, McGuire P. Real-world long-term outcomes in individuals at clinical risk for psychosis: The case for extending duration of care. EClinicalMedicine. 2020;28:100578.

5. Addington J, van der Gaag M. Psychosocial treatments for clinical high risk individuals. Schizophr Bull. 2015;41(1):22.

6. McGlashan TH. Early detection and intervention in schizophrenia: research. Schizophr Bull. 1996;22(2):327-45.

7. McGorry PD, Hartmann JA, Spooner R, Nelson B. Beyond the "at risk mental state" concept: transitioning to transdiagnostic psychiatry. World Psychiatry. 2018;17(2):133-42.

8. Polari A, Lavoie S, Yuen HP, Amminger P, Berger G, Chen E, et al. Clinical trajectories in the ultra-high risk for psychosis population. Schizophr Res. 2018;197:550-6.

9. Lin A, Wood SJ, Nelson B, Beavan A, McGorry P, Yung AR. Outcomes of nontransitioned cases in a sample at ultra-high risk for psychosis. Am J Psychiatry. 2015;172(3):249-58.

10. Fusar-Poli P, Nelson B, Valmaggia L, Yung AR, McGuire PK. Comorbid depressive and anxiety disorders in 509 individuals with an at-risk mental state: impact on psychopathology and transition to psychosis. Schizophr Bull. 2014;40(1):120-31.

11. Addington J, Piskulic D, Liu L, Lockwood J, Cadenhead KS, Cannon TD, et al. Comorbid diagnoses for youth at clinical high risk of psychosis. Schizophr Res. 2017;190:90-5.

12. Beck K, Andreou C, Studerus E, Heitz U, Ittig S, Leanza L, et al. Clinical and functional long-term outcome of patients at clinical high risk (CHR) for psychosis without transition to psychosis: A systematic review. Schizophr Res. 2019;210:39-47.

13. Fusar-Poli P, Cappucciati M, Borgwardt S, Woods SW, Addington J, Nelson B, et al. Heterogeneity of Psychosis Risk Within Individuals at Clinical High Risk: A Meta-analytical Stratification. JAMA Psychiatry. 2016;73(2):113-20.

14. Taylor PJ, Hutton P, Wood L. Are people at risk of psychosis also at risk of suicide and self-harm? A systematic review and meta-analysis. Psychol Med. 2015;45(5):911-26.

15. Schultze-Lutter F, Klosterkotter J, Michel C, Winkler K, Ruhrmann S. Personality disorders and accentuations in at-risk persons with and without conversion to first-episode psychosis. Early Interv Psychiatry. 2012;6(4):389-98.

16. Addington J, Case N, Saleem MM, Auther AM, Cornblatt BA, Cadenhead KS. Substance use in clinical high risk for psychosis: a review of the literature. Early Interv Psychiatry. 2014;8(2):104-12.

17. Chang WC, Ng CM, Chan KN, Lee HC, Chan SI, Chiu SS, et al. Psychiatric comorbidity in individuals at-risk for psychosis: Relationships with symptoms, cognition and psychosocial functioning. Early Interv Psychiatry. 2021;15(3):616-23.

18. Woods SW, Addington J, Cadenhead KS, Cannon TD, Cornblatt BA, Heinssen R, et al. Validity of the prodromal risk syndrome for first psychosis: findings from the North American Prodrome Longitudinal Study. Schizophr Bull. 2009;35(5):894-908.

19. Ryan J, Graham A, Nelson B, Yung A. Borderline personality pathology in young people at ultra high risk of developing a psychotic disorder. Early Interv Psychiatry. 2017;11(3):208-14.

20. Vines L, Bridgwater M, Bachman P, Hayes R, Catalano S, Jalbrzikowski M. Elevated emotion reactivity and emotion regulation in individuals at clinical high risk for developing psychosis and those diagnosed with a psychotic disorder. Early Interv Psychiatry. 2022;16(7):724-35.

21. Paust T, Theodoridou A, Müller M, Wyss C, Obermann C, Rössler W, et al. Borderline Personality Pathology in an At Risk Mental State Sample. Front Psychiatry. 2019;10:838.

22. West ML, Guest RM, Carmel A. Comorbid early psychosis and borderline personality disorder: Conceptualizing clinical overlap, etiology, and treatment. Personal Ment Health. 2021;15(3):208-22.

23. Boldrini T, Tanzilli A, Di Cicilia G, Gualco I, Lingiardi V, Salcuni S, et al. Personality Traits and Disorders in Adolescents at Clinical High Risk for Psychosis: Toward a Clinically Meaningful Diagnosis. Front Psychiatry. 2020;11:562835.

24. Sengutta M, Gawęda Ł, Moritz S, Karow A. The mediating role of borderline personality features in the relationship between childhood trauma and psychotic-like experiences in a sample of help-seeking non-psychotic adolescents and young adults. Eur Psychiatry. 2019;56:84-90.

25. Varese F, Smeets F, Drukker M, Lieverse R, Lataster T, Viechtbauer W, et al. Childhood adversities increase the risk of psychosis: a meta-analysis of patient-control, prospective- and cross-sectional cohort studies. Schizophr Bull. 2012;38(4):661-71.

26. Kraan T, Velthorst E, Smit F, de Haan L, van der Gaag M. Trauma and recent life events in individuals at ultra high risk for psychosis: review and meta-analysis. Schizophr Res. 2015;161(2-3):143-9.

27. Loewy RL, Corey S, Amirfathi F, Dabit S, Fulford D, Pearson R, et al. Childhood trauma and clinical high risk for psychosis. Schizophr Res. 2019;205:10-4.

28. WHO. Preventing suicide: A global imperative. Geneva2014.

29. Kelleher I, Corcoran P, Keeley H, Wigman JT, Devlin N, Ramsay H, et al. Psychotic symptoms and population risk for suicide attempt: a prospective cohort study. JAMA Psychiatry. 2013;70(9):940-8.

30. Kline ER, Seidman LJ, Cornblatt BA, Woodberry KA, Bryant C, Bearden CE, et al. Depression and clinical high-risk states: Baseline presentation of depressed vs. non-depressed participants in the NAPLS-2 cohort. Schizophr Res. 2018;192:357-63.

31. van Os J, Guloksuz S. A critique of the "ultra-high risk" and "transition" paradigm. World Psychiatry. 2017;16(2):200-6.

32. Carney R, Cotter J, Firth J, Bradshaw T, Yung AR. Cannabis use and symptom severity in individuals at ultra high risk for psychosis: a meta-analysis. Acta Psychiatr Scand. 2017;136(1):5-15.

33. Kraan T, Velthorst E, Koenders L, Zwaart K, Ising HK, van den Berg D, et al. Cannabis use and transition to psychosis in individuals at ultra-high risk: review and meta-analysis. Psychol Med. 2016;46(4):673-81.

34. Addington J, Cadenhead KS, Cornblatt BA, Mathalon DH, McGlashan TH, Perkins DO, et al. North American Prodrome Longitudinal Study (NAPLS 2): overview and recruitment. Schizophr Res. 2012;142(1-3):77-82.

35. Scott EM, Hermens DF, Naismith SL, Guastella AJ, White D, Whitwell BG, et al. Distress and disability in young adults presenting to clinical services with mood disorders. Int J Bipolar Disord. 2013;1:23.

36. Addington J, Stowkowy J, Cadenhead KS, Cornblatt BA, McGlashan TH, Perkins DO, et al. Early traumatic experiences in those at clinical high risk for psychosis. Early Interv Psychiatry. 2013;7(3):300-5.

37. Thompson AD, Nelson B, Yuen HP, Lin A, Amminger GP, McGorry PD, et al. Sexual trauma increases the risk of developing psychosis in an ultra high-risk "prodromal" population. Schizophr Bull. 2014;40(3):697-706.

38. Hutton P, Taylor PJ. Cognitive behavioural therapy for psychosis prevention: a systematic review and meta-analysis. Psychol Med. 2014;44(3):449-68.

39. Devoe DJ, Farris MS, Townes P, Addington J. Interventions and social functioning in youth at risk of psychosis: A systematic review and meta-analysis. Early Interv Psychiatry. 2019;13(2):169-80.

40. van der Gaag M, Smit F, Bechdolf A, French P, Linszen DH, Yung AR, et al. Preventing a first episode of psychosis: meta-analysis of randomized controlled prevention trials of 12 month and longer-term follow-ups. Schizophr Res. 2013;149(1-3):56-62.

41. Stafford MR, Jackson H, Mayo-Wilson E, Morrison AP, Kendall T. Early interventions to prevent psychosis: systematic review and meta-analysis. BMJ. 2013;346:f185.

42. Kuo JR, Khoury JE, Metcalfe R, Fitzpatrick S, Goodwill A. An examination of the relationship between childhood emotional abuse and borderline personality disorder features: the role of difficulties with emotion regulation. Child Abuse Negl. 2015;39:147-55.

43. Chapman HC, Visser KF, Mittal VA, Gibb BE, Coles ME, Strauss GP. Emotion regulation across the psychosis continuum. Dev Psychopathol. 2020;32(1):219-27.

44. Slee N, Garnefski N, Spinhoven P, Arensman E. The influence of cognitive emotion regulation strategies and depression severity on deliberate self-harm. Suicide Life Threat Behav. 2008;38(3):274-86.

45. Kimhy D, Lister A, Liu Y, Vakhrusheva J, Delespaul P, Malaspina D, et al. The impact of emotion awareness and regulation on psychotic symptoms during daily functioning. NPJ Schizophr. 2020;6(1):7.

46. Kimhy D, Gill KE, Brucato G, Vakhrusheva J, Arndt L, Gross JJ, et al. The impact of emotion awareness and regulation on social functioning in individuals at clinical high risk for psychosis. Psychol Med. 2016;46(14):2907-18.

47. Ludwig L, Werner D, Lincoln TM. The relevance of cognitive emotion regulation to psychotic symptoms - A systematic review and meta-analysis. Clin Psychol Rev. 2019;72:101746.

48. Conner KR, Pinquart M, Gamble SA. Meta-analysis of depression and substance use among individuals with alcohol use disorders. J Subst Abuse Treat. 2009;37(2):127-37.

49. Addington J, Addington D, Abidi S, Raedler T, Remington G. Canadian Treatment Guidelines for Individuals at Clinical High Risk of Psychosis. Can J Psychiatry. 2017;62(9):656-61.

50. Linehan MM, Korslund KE, Harned MS, Gallop RJ, Lungu A, Neacsiu AD, et al. Dialectical behavior therapy for high suicide risk in individuals with borderline personality disorder: a randomized clinical trial and component analysis. JAMA Psychiatry. 2015;72(5):475-82.

51. Harley R, Sprich S, Safren S, Jacobo M, Fava M. Adaptation of dialectical behavior therapy skills training group for treatment-resistant depression. J Nerv Ment Dis. 2008;196(2):136-43.

52. Safer DL, Robinson AH, Jo B. Outcome from a randomized controlled trial of group therapy for binge eating disorder: comparing dialectical behavior therapy adapted for binge eating to an active comparison group therapy. Behav Ther. 2010;41(1):106-20.

53. Goldstein TR, Fersch-Podrat RK, Rivera M, Axelson DA, Merranko J, Yu H, et al. Dialectical behavior therapy for adolescents with bipolar disorder: results from a pilot randomized trial. J Child Adolesc Psychopharmacol. 2015;25(2):140-9.

54. Neacsiu AD, Eberle JW, Kramer R, Wiesmann T, Linehan MM. Dialectical behavior therapy skills for transdiagnostic emotion dysregulation: a pilot randomized controlled trial. Behav Res Ther. 2014;59:40-51.

55. Lawlor C, Vitoratou S, Duffy J, Cooper B, De Souza T, Le Boutillier C, et al. Managing emotions in psychosis: Evaluation of a brief DBT-informed skills group for individuals with psychosis in routine community services. Br J Clin Psychol. 2022;61(3):735-56.

56. Kurdyak P, Zaheer J, Carvalho A, Oliveira Cd, Lebenbaum M, Wilton AS, et al. Physician-based availability of psychotherapy in Ontario: a population-based retrospective cohort study. CMAJ Open. 2020;8(1):E105-E15.

57. Bartram M, Stewart JM. Income-based inequities in access to psychotherapy and other mental health services in Canada and Australia. Health Policy. 2019;123(1):45-50.

58. Moroz N, Moroz I, D'Angelo MS. Mental health services in Canada: Barriers and cost-effective solutions to increase access. Healthc Manage Forum. 2020;33(6):282-7.

59. Gega L, Marks I, Mataix-Cols D. Computer-aided CBT self-help for anxiety and depressive disorders: experience of a London clinic and future directions. J Clin Psychol. 2004;60(2):147-57.

60. Reilly T, Mechelli A, McGuire P, Fusar-Poli P, Uhlhaas PJ. E-Clinical High Risk for Psychosis: Viewpoint on Potential of Digital Innovations for Preventive Psychiatry. JMIR Ment Health. 2019;6(10):e14581.

61. Wilks CR, Lungu A, Ang SY, Matsumiya B, Yin Q, Linehan MM. A randomized controlled trial of an Internet delivered dialectical behavior therapy skills training for suicidal and heavy episodic drinkers. J Affect Disord. 2018;232:219-28.

62. Saunders B, Sim J, Kingstone T, Baker S, Waterfield J, Bartlam B, et al. Saturation in qualitative research: exploring its conceptualization and operationalization. Qual Quant. 2018;52(4):1893-907.

63. Sebele-Mpofu FY. Saturation controversy in qualitative research: Complexities and underlying assumptions. A literature review. Cogent Social Sciences. 2020.

64. Spencer L, Ritchie, J., Ormston, R., O’Connor, W., and Barnard, M. . Qualitative research practice : a guide for social science students and researchers. 2nd ed. London: Sage Publications Ltd; 2014.

65. Tobin GA, Begley CM. Methodological rigour within a qualitative framework. J Adv Nurs. 2004;48(4):388-96.

66. Attkisson CC, Zwick R. The client satisfaction questionnaire. Psychometric properties and correlations with service utilization and psychotherapy outcome. Eval Program Plann. 1982;5(3):233-7.

67. Brooke J. SUS: a quick and dirty usability scale.: Research Gate 1995 [Available from: <https://www.researchgate.net/publication/228593520_SUS_A_quick_and_dirty_usability_scale>.

68. Carrion RE, Auther AM, McLaughlin D, Olsen R, Addington J, Bearden CE, et al. The Global Functioning: Social and Role Scales-Further Validation in a Large Sample of Adolescents and Young Adults at Clinical High Risk for Psychosis. Schizophr Bull. 2019;45(4):763-72.

69. Miller TJ, McGlashan TH, Rosen JL, Cadenhead K, Cannon T, Ventura J, et al. Prodromal assessment with the structured interview for prodromal syndromes and the scale of prodromal symptoms: predictive validity, interrater reliability, and training to reliability. Schizophr Bull. 2003;29(4):703-15.

70. Miller TJ, Zipursky RB, Perkins D, Addington J, Woods SW, Hawkins KA, et al. The PRIME North America randomized double-blind clinical trial of olanzapine versus placebo in patients at risk of being prodromally symptomatic for psychosis. II. Baseline characteristics of the "prodromal" sample. Schizophr Res. 2003;61(1):19-30.

71. Tluczek A, Henriques JB, Brown RL. Support for the reliability and validity of a six-item state anxiety scale derived from the State-Trait Anxiety Inventory. J Nurs Meas. 2009;17(1):19-28.

72. Addington D, Addington J, Maticka-Tyndale E. Assessing depression in schizophrenia: the Calgary Depression Scale. Br J Psychiatry Suppl. 1993(22):39-44.

73. Lawlor C, Vitoratou S, Hepworth C, Jolley S. Self-reported emotion regulation difficulties in psychosis: Psychometric properties of the Difficulties in Emotion Regulation Scale (DERS-16). J Clin Psychol. 2021;77(10):2323-40.

74. Robinson SM, Sobell LC, Sobell MB, Leo GI. Reliability of the Timeline Followback for cocaine, cannabis, and cigarette use. Psychol Addict Behav. 2014;28(1):154-62.

75. Adamson SJ, Kay-Lambkin FJ, Baker AL, Lewin TJ, Thornton L, Kelly BJ, et al. An improved brief measure of cannabis misuse: the Cannabis Use Disorders Identification Test-Revised (CUDIT-R). Drug Alcohol Depend. 2010;110(1-2):137-43.

76. Cuttler C, Spradlin A. Measuring cannabis consumption: Psychometric properties of the Daily Sessions, Frequency, Age of Onset, and Quantity of Cannabis Use Inventory (DFAQ-CU). PLoS One. 2017;12(5):e0178194.

77. Kleindienst N, Jungkunz M, Bohus M. A proposed severity classification of borderline symptoms using the borderline symptom list (BSL-23). Borderline Personal Disord Emot Dysregul. 2020;7(1):11.

78. Connor KM, Davidson JR. Development of a new resilience scale: the Connor-Davidson Resilience Scale (CD-RISC). Depress Anxiety. 2003;18(2):76-82.

79. Posner K, Brown GK, Stanley B, Brent DA, Yershova KV, Oquendo MA, et al. The Columbia-Suicide Severity Rating Scale: initial validity and internal consistency findings from three multisite studies with adolescents and adults. Am J Psychiatry. 2011;168(12):1266-77.

80. Nuechterlein KH, Green MF, Kern RS, Baade LE, Barch DM, Cohen JD, et al. The MATRICS Consensus Cognitive Battery, part 1: test selection, reliability, and validity. Am J Psychiatry. 2008;165(2):203-13.

81. Wisniewski H, Torous J. Digital navigators to implement smartphone and digital tools in care. Acta Psychiatr Scand. 2020;141(4):350-5.

82. Neacsiu AD, Rizvi SL, Vitaliano PP, Lynch TR, Linehan MM. The dialectical behavior therapy ways of coping checklist: development and psychometric properties. J Clin Psychol. 2010;66(6):563-82.

83. Julious SA. Sample size of 12 per group rule of thumb for a pilot study. Pharmaceut Sta. 2005;4:287–91.

84. Sim J, Lewis M. The size of a pilot study for a clinical trial should be calculated in relation to considerations of precision and efficiency. J Clin Epidemiol. 2012;65(3):301-8.

85. Teare MD, Dimairo M, Shephard N, Hayman A, Whitehead A, Walters SJ. Sample size requirements to estimate key design parameters from external pilot randomised controlled trials: a simulation study. Trials. 2014;15:264.

86. First MB, Spitzer RL, Gibbon M, Williams JB. Structured clinical interview for DSM-IV-TR axis I disorders, research version, patient edition. SCID-I/P New York, NY, USA:; 2002.

87. Addington J, Shah H, Liu L, Addington D. Reliability and validity of the Calgary Depression Scale for Schizophrenia (CDSS) in youth at clinical high risk for psychosis. Schizophr Res. 2014;153(1-3):64-7.

88. Cornblatt BA, Auther AM, Niendam T, Smith CW, Zinberg J, Bearden CE, et al. Preliminary findings for two new measures of social and role functioning in the prodromal phase of schizophrenia. Schizophr Bull. 2007;33(3):688-702.

89. Moberg DP. Screening for alcohol and other drug problems using the Adolescent Alcohol and Drug Involvement Scale (AADIS). . Madison, Wisconsin USA: Center for Health Policy and Program Evaluation, University of Wisconsin--Madison; 2003.

90. Alvarez-Jimenez M, Gleeson JF, Bendall S, Penn DL, Yung AR, Ryan RM, et al. Enhancing social functioning in young people at Ultra High Risk (UHR) for psychosis: A pilot study of a novel strengths and mindfulness-based online social therapy. Schizophr Res. 2018;202:369-77.

91. Henderson J, Courey L, Relihan J, Darnay K, Szatmari P, Cleverley K, et al. Youth and family members make meaningful contributions to a randomized-controlled trial: YouthCan IMPACT. Early Interv Psychiatry. 2021.
